# *In vitro* Targetability Validation of Peptide-Functionalized Mesoporous Silica Nanoparticles in the Presence of Serum Proteins

**DOI:** 10.3389/fchem.2020.603616

**Published:** 2020-11-13

**Authors:** Valeriy M. Paramonov, Melanie Gerstenberg, Cecilia Sahlgren, Mika Lindén, Adolfo Rivero-Müller

**Affiliations:** ^1^Institute of Biomedicine, Research Centre for Integrative Physiology and Pharmacology, University of Turku, Turku, Finland; ^2^Turku Bioscience, University of Turku and Åbo Akademi University, Turku, Finland; ^3^Faculty of Science and Engineering, Cell Biology, Åbo Akademi University, Turku, Finland; ^4^Department of Inorganic Chemistry II, Ulm University, Ulm, Germany; ^5^Institute for Complex Molecular Systems, Eindhoven University of Technology, Eindhoven, Netherlands; ^6^Department of Biochemistry and Molecular Biology, Medical University of Lublin, Lublin, Poland

**Keywords:** nanoparticle, targetability, nanoparticle corona, mesoporous silica, somatostatin receptor, cAMP

## Abstract

Demonstration of receptor-mediated targeting of nanoparticles to specific organs and/or cell types is an integral aim in many bionanomedicine development projects. However, engagement of targeted receptors with ligands on nanocarriers, which is the cornerstone of the active targeting concept, is challenging to study under biologically relevant conditions and thus often stays overlooked. In this work, we utilize an in-house established bioassay for *in vitro* targetability validation of mesoporous silica nanoparticles (MSNs), functionalized with high-affinity peptide ligands to somatostatin receptors via protective group chemistry, ensuring the correct orientation of the peptide's pharmacophore. We demonstrate that targeted nanoparticles, but not scrambled peptide-decorated counterparts, specifically engage the targeted receptors in living cells in culture media containing serum protein. The importance of being able to exclude false positives originating from the premature detachment of targeting peptides from the MSNs is highlighted.

## Introduction

Nanoparticulate drug carriers promise to address several bottlenecks of conventional systemic therapy, including suboptimal pharmacokinetics, issues with biodistribution, clearance, drug stability, and toxicity (Mamaeva et al., 2013; Shi et al., 2017; Wolfram and Ferrari, 2019). High hopes are specifically placed on the so-called active targeting concept, where nanocarriers are functionalized with high-affinity ligands to receptors of high abundance in diseased tissues. Specific binding of the targeted ligands attached the nanocarriers to the receptors in question is expected to enhance the site-specific accumulation of the therapeutic cargo at the target site (Sikorski et al., 2015; Rosenblum et al., 2018; Yoo et al., 2019). Validation of active targeting, i.e., specific binding of targeted receptors by nanoparticle-anchored ligands, is, however, not straightforward. Typical approaches for demonstrating active *in vitro* targeting in published studies rely on comparative assays with two cell lines with differential expression of a targeted receptor or on competition experiments with a free targeting ligand. Both of these methods suffer from important limitations, as cell lines under comparison often have different metabolic rates, which could also be affected by the addition of a free ligand. Besides, experiments to validate the engagement of targeted receptors with targeting ligands on nanoparticles tend to be substituted with indirect methods, inferring targetability from subsequent events, such as the differential uptake of nanoparticles by cells or alterations in cellular phenotypes upon study treatments. Thus, the direct proof of active targeting is often missing (Paramonov et al., 2018).

We have earlier developed an *in vitro* bioassay for the validation of active targeting, which allows for kinetic analysis of receptor engagement in living cells by targeting ligands on nanocarriers. The bioassay utilizes a genetically encoded luminescent sensor for 3′-5′-cyclic adenosine monophosphate (cAMP) and enables quantification of ligand-induced activation of G protein-coupled receptors (GPCRs), signaling via cAMP (Paramonov et al., 2018). More specifically, the bioassay is based on HEK293 cells stably co-expressing GloSensor-22F cAMP probe and a selected subtype of somatostatin receptors (SSTR2, 3 or 5). GloSensor-22F, originally introduced by Wood and co-authors (Fan et al., 2008; Binkowski et al., 2011), is a fast, reversible, and sensitive cAMP probe, based on *Photinus pyralis* luciferase fused with IIβ subunit of protein kinase A. SSTRs belong to the GPCR superfamily and act as negative regulators of adenylyl cyclases (ACs), which are cAMP-generating enzymes. Thus, SSTR activation with cognate ligands, be it free peptides or peptides, anchored to the MSN surface, results in a drop of intracellular cAMP, which is captured by the GloSensor-22F probe. Thereby, the sensor cells comprise an all-in-one system for the quantitative measurement of SSTR signaling. To facilitate the detection of cAMP decrements, basal levels of intracellular cAMP are shifted upwards by means of forskolin (FSK), a potent activator of ACs, which is co-administered to the sensor cells together with the study treatments.

In our previous work we validated the *in vitro* targetability of a set of mesoporous silica nanoparticles (MSNs), functionalized with peptide ligands of SSTRs (Paramonov et al., 2018). Although the results indicated that the MSNs could be actively targeted, the study was performed under protein-depleted conditions (culture medium with 0.1%BSA), and the effects of nanoparticle corona of serum opsonins on MSN targetability were not addressed. Furthermore, the potential influence of premature detachment of targeting peptides from the MSNs in culture medium was not evaluated.

In the present study, we utilize similar nanoparticles as in the earlier work (MSNs with mean diameter of ≈180 nm in a dry state) and the same targetability mode (peptides for targeting ligands and SSTRs for targeted receptors). We assess targetability in a more realistic biological setting, implementing our targetability bioassay under serum-enriched conditions to evaluate the effects of MSN opsonization (protein corona formation) on the ability of MSNs to engage the targeted receptors. Moreover, in view of the limitations of the earlier-utilized peptide functionalization protocols, characterized by the low efficiency of the peptide attachment and suboptimal control over the directionality of the peptide anchoring to the MSN surface (Paramonov et al., 2018), we implement and evaluate three alternative methods for peptide linking, utilizing protective group chemistry during peptide synthesis and attachment, in order to ensure correct peptide orientation.

Our studies reveal functional differences between peptide linking chemistries, manifested as varying rates of peptide shedding from the MSN surface upon MSN passage from the monocomponent aqueous buffer to the complex culture medium. Through a careful evaluation of MSN supernatants and non-fractionated MSN preps, we deduce the genuine input of MSN-anchored peptides in the net receptor activation, thus validating MSN targetability in protein-depleted and, most importantly, serum-enriched media.

## Materials and Methods

### Compounds and Reagents

Diisopropylethylamine (DiPEA), triisopropylsilane chloride (TiPS), tetramethoxysilane (TMOS), (3-aminopropyl)trimethoxysilane (APTMS), N-(3-(dimethylamino)propyl)-N′-ethylcarbodiimide (EDC), N-hydroxysuccinimide (NHS), triethylamine, piperidine, and glycerin were obtained from Sigma Aldrich Chemie GmbH (Munich, Germany). Fmoc and side chain-protected amino acids, ammonium nitrate, and 2-[4-(2-hydroxyethyl)-1-piperazinyl]ethane sulfonic acid sodium salt (HEPES) potassium chloride were purchased from Merck KGaA (Darmstadt, Germany). H-Threoninol(But)-2-ClTrt-resin was obtained from Advanced ChemTech (Louisville, USA). Tetramethylethylendiamin (TEMED), dimethylformamide (DMF), cetyltrimethylammonium bromide (CTAB), methanol, and sodium hydroxide were obtained from VWR International GmbH (Radnor, USA) and ATTO647N-amine from ATTO-TEC GmbH (Siegen, Germany). 2-(1H-benzotriazol-1-yl)-1,1,3,3-tetramethyluroniumhexafluoro-phosphate (HBTU) was bought at Carbolution Chemicals GmbH (Saarbrücken, Germany) and trifluoroacetic acid (TFA), acrylamide (≥98%), agarose (low melt), ammonium peroxodisulfate (≥98%), Coomassie® Brilliant Blue G-250, glycine (≥99%), N,N′-Methylen-bis-acrylamide (≥98%), and sodium dodecyl sulfate (SDS) were obtained from Carl Roth GmbH & Co. KG (Karlsruhe, Germany). PageRuler unstained protein ladder, dimethylsulfoxide (DMSO), Dulbecco's modified Eagle's medium (DMEM), fetal calf serum (FCS), and Dulbecco's phosphate-buffered saline (PBS, without calcium and magnesium) were purchased from Thermo Fisher Scientific Inc. (Waltham, USA). Carboxyethylsilanetriol sodium salt was obtained from abcr GmbH (Karlsruhe, Germany). Somatostatin−14 (#H-1490) and octreotide acetate (#H-5972) were obtained from Bachem (Switzerland). The peptides were kept at −80°C as single-use 100 μM aliquots. FSK was from LC laboratories, USA (#F-9929) and kept aliquoted (10 mM) in DMSO at −20°C. Triton X-100 was purchased from Sigma (#93443). All the chemical compounds and reagents used in the study were dissolved in ultrapure water (Milli-Q; resistivity >18 mΩ^*^cm), if not specified otherwise.

### Peptide Synthesis

The peptides F*CFW*KTC-threoninol (OC) and F*CFAATC-threoninol (SP) were synthesized using Fmoc solid-phase synthesis and threoninol-functionalized resin. D-amino acids (denoted with ^*^) were coupled with collidin instead of DiPEA to prevent racemization. The fully protected peptides were cleaved off the support through treatment with 1% TFA in dichloromethane for 1 h at RT. The lyophilized peptides were analyzed by mass spectroscopy on a Bruker solariX Hybrid 7T FT-ICR (Bruker, USA).

### Synthesis of MSNs

MSNs were synthesized through a modified synthesis, described by Rosenholm et al. (2009). In brief, cetyltrimethylammonium bromide, CTAB, (7.90 g; 21.7 mmol), that was used as the structure-directing agent, was dissolved in a mixture of methanol (640.0 g; 19.98 mol) and water (962.3 g; 53.43 mol), containing NaOH (4.56 mL; 1 M; 4.56 mmol). A mixture of TMOS (2.18 mL; 13.9 mmol) and APTMS (360.0 μL; 1.955 mmol) was added to this solution under continuous stirring. The resulting solution was allowed to react for 24 h at RT, after which the particles were separated by centrifugation (10,000 rpm, 10 min.). The surfactant was removed by calcination at 550°C for 6 h (at increment of +1°C/min till 550°C and then for 6 h at 550°C).

### MSN Functionalization With Targeting Peptides

The surface of the MSN was modified by covalent attachment of a silane, carrying a carboxylate group. MSNs were dispersed in a 1:1-mixture of deionized water and methanol (10.0 mg/mL), containing sodium hydroxide (45.6 μL; 1 M; 45.6 μmol), followed by addition of carboxyethylsilanetriol sodium salt (25% in water; 2.3 μL/mg; 2.9 μmol/mg) under stirring. For MSN_OC1_ and MSN_SP1_, peptide-functionalized carboxyethylsilanetriol was used. After a reaction time of 3 h at RT, the particles were separated by centrifugation (10,000 rpm, 10 min) and washed once with water and two times with methanol. The particles were subsequently dried in vacuum at RT.

#### MSN_OC1_/MSN_SP1_

To functionalize the carboxyethylsilanetriol sodium salt with peptides, EDC/NHS activation was used. Therefore, EDC (0.9 mg/μL; 4.6 μmol/μL) and NHS (0.6 mg/μL; 4.8 μmol/μL) were added to carboxyethylsilanetriol sodium salt (25% in water) and rotated for 30 min at RT. The protected peptides were dissolved in methanol (44.4 mg/mL for OC; 35.6 mg/mL for SP; 25 μmol/mL). The resulting peptide solutions were added to the activated carboxyethylsilanetriol sodium salt (1.0 μL/μL; 25 μmol/mL) and rotated for 2 h at RT. MSNs were dispersed in a 1:1-mixture of deionized water and methanol (10.0 mg/mL), containing sodium hydroxide (45.6 μL; 1 M; 45.6 μmol), with subsequent addition of peptide - functionalized carboxyethylsilanetriol sodium salt (12.5% in water; 4.0 μL/mg; 2.9 μmol/mg). After 3-h rotation at RT, MSNs were separated by centrifugation (10,000 rpm, 10 min) and washed once with water and two times with methanol. The resulting carboxylized particles were dried in vacuum at RT. The amount of MSN-coupled peptides was estimated via UV/Vis measurements of the stock solutions and the supernatants on NanoDrop 2000c (Thermo Fisher Scientific, USA).

#### MSN_OC2_/MSN_SP2_ and MSN_OC3_/MSN_SP3_

For peptide coupling, the carboxy-functions were activated with 1-ethyl-3-(3-dimethylaminopropyl)carbodiimide, EDC, (2.0 mg EDC/mg MSNs; 10 μmol/mg) and N-hydroxysuccinimide, NHS, (1.25 mg NHS/mg MSN; 10.9 μmol/mg) in deionized water (10 mg MSN/mL) for 30 min at RT, followed by washing with water. MSNs were then re-dispersed in methanol (OC2 and SP2) or DMF (OC3 and SP3), to 20 mg of MSN/mL. The protected peptide was dissolved in methanol or DMF (2.0 mg peptide/mL for OC; 1.6 mg peptide/mL for SP; 1.1 μmol/mL). The particle dispersions were diluted 1:1 with the peptide solutions and rotated for 20 h at RT. The particles were subsequently washed once with methanol or DMF and two times with methanol. MSN drying and peptide load measurements were carried out, as specified above.

#### Further Functionalization of all MSN Types

The protected peptide-functionalized MSNs were further covalently labeled with ATTO647N dye. The particles were dispersed in HEPES buffer solution (10 mg MSN/mL). For EDC/NHS activation of the carboxy groups, EDC (1.7 μL/mg MSN; 9.6 μmol/mg) and NHS (1.15 μg/mg MSN; 9.99 μmol/mg) were added to the particle dispersion. After 30-min rotation at RT, the particles were washed once with HEPES buffer (25 mM, pH 7.2). Thereafter, the particles were re-dispersed in HEPES buffer (25 mM, pH 7.2; 10 mg MSN/mL), containing ATTO647N-amine/DMSO stock solution (1 mg/mL; 18 μg dye/mg MSN; 2.0 nmol/mg MSN). After further rotation for 1 h at RT under exclusion of light, the particles were washed three times with methanol and dried under vacuum at RT.

Finally, in order to de-protect the side chains of the attached peptides, the particles were dispersed in 95% trifluoroacetic acid with 5% water and 5% triisopropylsilane (10 mg MSN/mL). After 2 h rotation at RT, the particles were washed twice with diethylether. Dried particles were dispersed in 5% acetic acid (pH = 6.0), containing 20% DMSO to cyclize the OC peptides by forming a disulfide bridge. For SP, DMSO was replaced by 5% acetic acid in order to prevent cyclisation. The dispersion was rotated for 4 h at RT, followed by a single wash with water and two washes with methanol. The final MSN preps were dried under vacuum at RT.

### Physicochemical Characterization of MSNs

Dynamic light scattering and zeta potential measurements were performed on Zetasizer Nano-ZS ZEN 3600 (Malvern Instruments, Great Britain) at 0.1 mg/mL of MSNs in 1 mM KCl solution. Nitrogen sorption measurements were performed at −196°C on a Quadrasorb-SI (Quantachrome Instruments, USA). The pore size and pore volume were determined via the calculation, based on the non-local density functional theory, using the kernel developed for silica materials with cylindrical mesopores. MSN size was determined via transmission electron microscopy (TEM) on a Joel 1400 (Joel, Germany), using an acceleration voltage of 120 kV. The amount of carboxy groups on MSNs was determined by thermogravimetric analysis (TGA) on Netzsch TG209 Libra F1 (Netzsch, Germany; heating rate = 10 K/min). The specific fluorescence intensities of the dye-labeled MSNs were determined on Infinite M1000 platereader (Tecan, Switzerland; ATTO647N: λ_exc_ = 635 nm; λ_em_ = 680 nm).

### MSN Protein Adsorption Analysis With SDS-PAGE

For protein adsorption analysis, MSNs were firstly dispersed in HEPES buffer (25 mM, pH 7.2; 5.0 mg MSN/mL) and then further diluted to 1.5 mg/ml in DMEM (with 10% heat-inactivated FCS and 1% Pen/Strep). This resulted in a final FCS concentration of 7.0%. After incubation at 37°C for 60 min, MSNs were washed three times with deionized water. For protein desorption, MSNs were dispersed in SDS buffer (10% SDS in deionized water; 10 mg MSN/mL) and treated on ultrasonic bath for 30 min at RT. After MSN sedimentation by centrifugation (1,480 rpm, 10 min), the retrieved supernatants were mixed with Laemmli buffer (20 μL sample + 4 μL Laemmli buffer); 10% SDS, 7% iFCS and PageRuler unstained protein ladder were prepared as controls.

One-dimensional sodium dodecyl sulfate-polyacrylamide gel electrophoresis (SDS-PAGE; 12% separating gel; 5% stacking gel) was performed in a Bio-Rad PROTEAN II XL electrophoresis chamber using constant 300 V for 1,066 Vh. The gels were washed four times with deionized water before staining the proteins for 2 h in Coomassie staining solution, as described by Kang et al. (2002). After destaining in deionized water for 24 h at RT, the gels were imaged on a GelDoc System and analyzed with the Image Lab software (both from Bio-Rad).

### MSNs Handling for Biological Tests

All suspensions of MSNs were prepared fresh immediately before experiments via a uniform procedure from the lyophilized stock, which was kept as small single-use pre-weighted aliquots at −20°C. After removal from the freezer and brief centrifugation to collect all the particulate material, an aliquot of MSNs was re-suspended in HEPES buffer (25 mM, pH 7.4) of RT to the desired concentration by vigorous vortexing and further processed on a waterbath sonicator (FinnSonic m03; FinnSonic Oy, Finland) for three rounds of 10 min each, with additional vortexing in between. The sonication was done in deionized H_2_0 of <10°C, with water temperature controlled by timely addition of regular ice. Before usage, the resulting MSN preps were further diluted in desired solvent to 10 × of the final working concentration. For the preformed corona studies, the freshly sonicated suspensions of MSNs were further diluted to 1,500 μg/ml in either DMEM/F-12 mix with 10% (w/v) of iFBS [corona arm; final serum concentration during incubation – 7% (w/v)] or HEPES buffer (25 mM, pH 7.4; control arm), with subsequent incubation for 1 h on a thermoshaker (Thermomixer Comfort, Eppendorf AG, Germany) at 850 RPM and +37°C. After the incubation, MSNs were further diluted to 10 × of the final working concentration in the same corresponding solvent (i.e., HEPES or medium+10% iFBS) and used immediately. For collection of supernatants (SNs), MSNs and controls were spun at 15,650×*g* for 5 min at RT, after which SN fractions were carefully removed by pipetting and transferred to fresh tubes.

### Cell Lines

Human embryonic kidney cell line HEK293 was obtained from American Type Culture Collection (ATCC, #CRL-1573). Human breast carcinoma cell line MCF7 was a kind gift from Prof. Urban Lendahl (Karolinska Institutet, Stockholm, Sweden). HEK293 with stable overexpression of GloSensor-22F cAMP sensor (aka HEK-GS), as well as the derived cells with stable overexpression of SSTR2, 3, and 5 (aka HEK-GS/SSTR2_HA, HEK-GS/SSTR3_Myc and HEK-GS/SSTR5_Flag, respectively), were developed and characterized by us earlier (Trehan et al., 2014; Paramonov et al., 2018). HEK293 wild-type cells and the derived strains were cultured in Dulbecco's Modified Eagle's Medium/Nutrient Mixture F-12 (DMEM/F-12; Gibco, #11320033). MCF7 was maintained in DMEM (Sigma, #D6171). The media were supplemented with 10% (w/v) of heat-inactivated fetal bovine serum (iFBS; Biowest, #S1810), 50 U/ml of penicillin, and 50 μg/ml streptomycin (Gibco, #15140122). The cells were maintained at +37°C in humidified atmosphere with 5% CO_2_. All cell counts were done with the TC20 automated cell counter (Bio-Rad Labs, USA).

### Scrambled Peptide *in vitro* Toxicity Screen

To exclude possible toxicity of SP at the intended dosing levels, we resorted to Cell Counting Kit-8 assay (CCK-8; Dojindo Europe, #CK04-13), which employs mitochondrial respiration-derived colorimetric readout for proxy of cell viability. CCK-8 was implemented after the vendor's suggestions, as follows. HEK-GS/SSTR2_HA and MCF7 cells were seeded in 96-well plates (Greiner, #655180) as 15,000 cells per well in 95 μl of the cell type-specific complete medium w/o antibiotics, and the plates were left overnight (ON) in the incubator. The next day, w/o prior medium exchange, the cells were spiked with 10 μl/well of 10× solutions of SP or corresponding controls (all prepared in 25 mM HEPES, pH 7.4), yielding desired 1× working concentrations, and the plates were returned to the incubator. Two hours before the assay's termination points, i.e., 24 and 48 h of treatment, 10 μl of CCK-8 reagent was added to the wells w/o prior medium exchange, and the plates were incubated further till the specified time points had been reached. The assay was terminated with absorbance read at 450 nm (Abs@450 nm; with EnSight multimodal plate reader, PerkinElmer, USA). After subtraction of the average blank values (corresponding to Abs@450 nm of cell-specific medium w/o cells), the resulting values were normalized to average Abs@450 nm of non-treated cells (taken for 100%), giving the final normalized viability rate.

### *In vitro* Targetability Bioassay: Measuring Intracellular cAMP in Living Cells With the Luminescent cAMP Probe

The experimental protocol and approaches to data processing are detailed in our earlier work (Paramonov et al., 2018). In brief, the cells with stable expression of GloSensor-22F cAMP probe (sensor cells) were seeded 1 day before the experiment into tissue culture-treated polystyrene 96-well plates with light-tight walls and translucent bottom (ViewPlate-96, PerkinElmer, Cat#6005181) as 60,000 cells per well in the 150 μl of cell type-specific medium, and incubated ON (+37°C in humidified atmosphere with 5% CO_2_). The next day, before the assay, the old culture medium was removed and the wells were refilled with 45 μl of the freshly prepared inducing medium (IndMed), composed of 2% v/v of GloSensor reagent (Promega, #E1290; corresponds to the final working concentration of 0.612 mg/ml, with the original stock of 30,6 mg/ml in 10 mM HEPES, pH 7.5) and 200 μM of a non-specific phosphodiesterase inhibitor 3-Isobutyl-1-methylxanthine (IMBX; Sigma, #I5879) in the assay-specific medium. In case of MSN corona studies, DMEM/F-12 medium (50/50, v/v) with 10% (w/v) of iFBS was used for that purpose (aka Med_10%FBS_, yielding IndMed_10%FBS_); for other setups, a mix of the above medium and CO_2_-independent medium (Gibco, #18045-054; 4v of DMEM/F12 per 5v of CO_2_-independent medium), supplemented with 0.1% (w/v) of bovine serum albumin (BSA), was used (aka Med_0.1%BSA_, yielding IndMed_0.1%BSA_). After equilibration for 45 min at RT in the dark, the plate was inserted into a multiwell plate reader (EnSight, PerkinElmer, USA) and the light output—denoted as a *baseline signal*—was captured in a kinetic fashion, i.e., the selected wells on a plate were repeatedly captured in a desired sequence, for 15–20 min at RT. Next, the plate was removed from the reader and the wells were spiked with either 5 μl of freshly prepared solutions, having all the desired components at 10× of the final concentration, or respective controls. Final concentration of FSK in the assay equaled 10 μM, if not specified otherwise. FSK was not subjected to heat exposure or centrifugation (in the context of peptide/MSN thermal stability or peptide shedding assays, respectively), but its working solutions were prepared simultaneously with the actual study preps and kept for the same time at RT before being mixed with the actual preps to yield the final 10× co-mixes. After spiking, the plate was immediately re-inserted into the reader and the luminescence (now denoted as *induced signal*) was further captured in the same kinetic mode for the time required (typically, for 45–60 min). The described assay conditions (i.e., at RT, IndMed with 2% of GloSensor reagent and 200 μM of IBMX, stimulation with 10 μM of FSK) are referred to as “standard” throughout the text.

The registered luminescent reads were used for plotting of intracellular cAMP kinetic curves (luminescence vs. time), with the latter processed to baseline signal—subtracted area under the curve (AUC) values with the help of a custom-written script (available from the authors upon request). The derived AUC values were further normalized to the AUC of FSK, taken for 100% (if not specified otherwise), and the resulting %FSK-AUC indices were used for inferential statistics.

### Data Transformation, Curve Fitting, and Statistics

Data transformations and all the statistical tests were carried out with the GraphPad Prism v8.3.0 package (GraphPad Software, San Diego, CA). Dose–response curves fitting and IC_50_ calculations were performed using ≪*log (inhibitor)* vs. *response - Variable slope*≫ (Y = Bottom + (Top − Bottom)/{1 + 10^∧^[(LogIC_50_ − X)* *HillSlope*]}) operator of GraphPad Prism software. Comparisons of dose effects of nanoparticles and the derived SNs were performed with either a repeated-measures ANOVA with mixed-effects model and Tukey's multiple comparisons test or with a paired one-tailed *t*-test (for number of groups under comparison ≥3 or 2, respectively). Level of significance was set to <0.05 for all of the tests [on the figures, one (*), two (**) and three (***) asterisks indicate *p*-values in the following ranges: [0.01; 0.05), [0.001; 0.01) and <0.001].

## Results

### Synthesis and Characterization of MSNs

**Figure 1:**
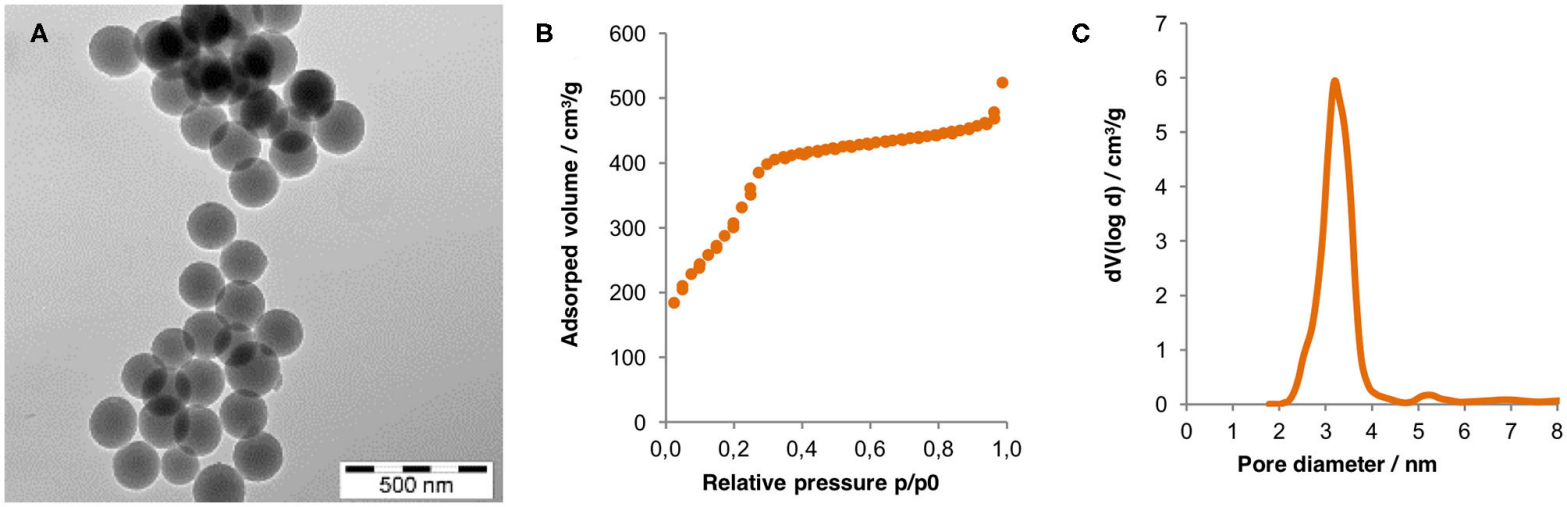
**(A)** TEM image, **(B)** nitrogen sorption isotherm, and **(C)** pore size distribution calculated via non-local functional theory (NLDFT) of the calcinated MSNs. Zeta potential and the hydrodynamic radius (both measured in 1 mM KCl, pH = 5.5) equaled −12 mV and 245 nm, respectively, highlighting the absence of particle aggregates after synthesis and calcination.

MSNs with a narrow particle size distribution and a mean diameter of ≈180 nm were synthesized using established methods. A transmission electron microscopy (TEM) image of the particles after removal of the porogen by calcination is shown on Figure 1. Also included is the nitrogen sorption isotherm measured for the same particles. The pore filling step within a narrow relative pressure range centered around 0.25 p/p_0_ gives support for a narrow mesopore size distribution. The mean mesopore diameter as calculated using the non-local density functional theory (NLDFT) equilibrium kernel, developed for silica and assuming a cylindrical pore geometry, was 3.1 nm. The specific surface area of the MSNs as calculated according to the Brunauer-Emmet-Teller (BET) theory, was 1,032 m^2^/g. These values are in full agreement with literature values (Beck et al., 1992; Mandal et al., 2018).

### Synthesis and Characterization of Peptide Ligands

In order to obtain a negative control for octreotide (_1D_Phe-[Cys-Phe-_D_Trp-Lys-Thr-Cys]-_8_Threoninol; OC), which was selected as the active targeting ligand in the study, we decided (1) to substitute D-Trp4 and Lys5 of the original OC sequence with Ala moieties and (2) to keep the resulting peptide linear by preventing Cys2–Cys7 bond formation (_1*D*_Phe*-*Cys-Phe-Ala-Ala-Thr-Cys-_8_Threoninol). Earlier structure-function studies highlight Phe3, D-Trp4, Lys5, and Thr6 as the essential residues, comprising the pharmacophore of OC, which is further stabilized by peptide cyclization through the mentioned disulfide bond (Vale et al., 1978; Bauer et al., 1982; Janecka et al., 2001). Thus, the designed structural alterations were expected to result in loss of its specific affinity for SSTRs, yielding a structurally comparable yet targeting-incapable scrambled peptide (SP).

OC and SP were synthesized by solid phase peptide synthesis. To ensure controlled binding of the peptides with the primary α-amino acid function, the peptides were cleaved from the resin in a fully protected form. Therefore, a threoninol-functionalized trityl resin and 1% TFA in dichloromethane were used as a cleavage cocktail. The acid strength was strong enough to cleave the linker between peptides and the resin, but not strong enough to de-protect the side chains of the synthesized peptides. Expected molecular weights of the fully protected peptides were 1,817 Da for the OC and 1,445 Da for the SP; purity of the synthesized peptides was confirmed by matrix assisted laser desorption ionization–time of flight mass spectrometry (MALDI-TOF MS; Supplementary Figure 1).

Before SP could be utilized for functionalization of the control MSNs, we had to ensure that the introduced structural alterations indeed rendered the peptide unable to engage SSTRs. To this end, we resorted to the dose-range studies of the de-protected SP with the targetability bioassay with HEK293 sensor cells, overexpressing SSTR2, 3, or 5. Importantly, the bioassay was performed with strict adherence to matched parallel design, with every analyte/targeted MSN dose compared to the matched dose of solvent/non-targeted MSNs in the same experiment. This minimizes variation and allows exposing the genuine effects of the experimental treatment.

Dose-matched studies of SP vs. its solvent, dimethylformamide (DMF), confirmed that SP lacked agonistic activity toward SSTR2, 3, and 5 across the dose range of 100 pM−10 μM (Supplementary Figures 2A–F). However, this evidence did not exclude the possibility of a silent receptor binding, i.e., ability of SP to specifically bind SSTRs without inducing receptor activation. To address this, SP was further characterized with the same types of sensor cells in a competition assay with somatostatin-14 (Sst14), which is an endogenous high-affinity agonist of all five SSTR subtypes (Paramonov et al., 2018). The addition of a high SP dose (1 μM) did not alter the pattern and potency of Sst14 response (Supplementary Figures 3A–G), which in conjunction with the earlier demonstrated absence of SSTR agonistic activity verifies that SP does not have a significant specific affinity toward SSTR2, 3, or 5.

Finally, to exclude SP toxicity, we performed a mitochondrial respiration-based cell viability screen in two unrelated cell lines, a human embryonic kidney cell line with overexpression of SSTR2 and the cAMP probe (HEK-GS/SSTR2_HA, also utilized as the sensor cells in the targetability bioassay) and a human breast carcinoma cell line (MCF7). SP at concentrations up to 10 μM did not affect the viability of the cells in question even after 48 h of treatment (Supplementary Figures 4A,B). Collectively, the data depict SP as a valid negative control ligand for MSN functionalization.

### MSN Functionalization With Targeting Ligands

**Figure 2:**
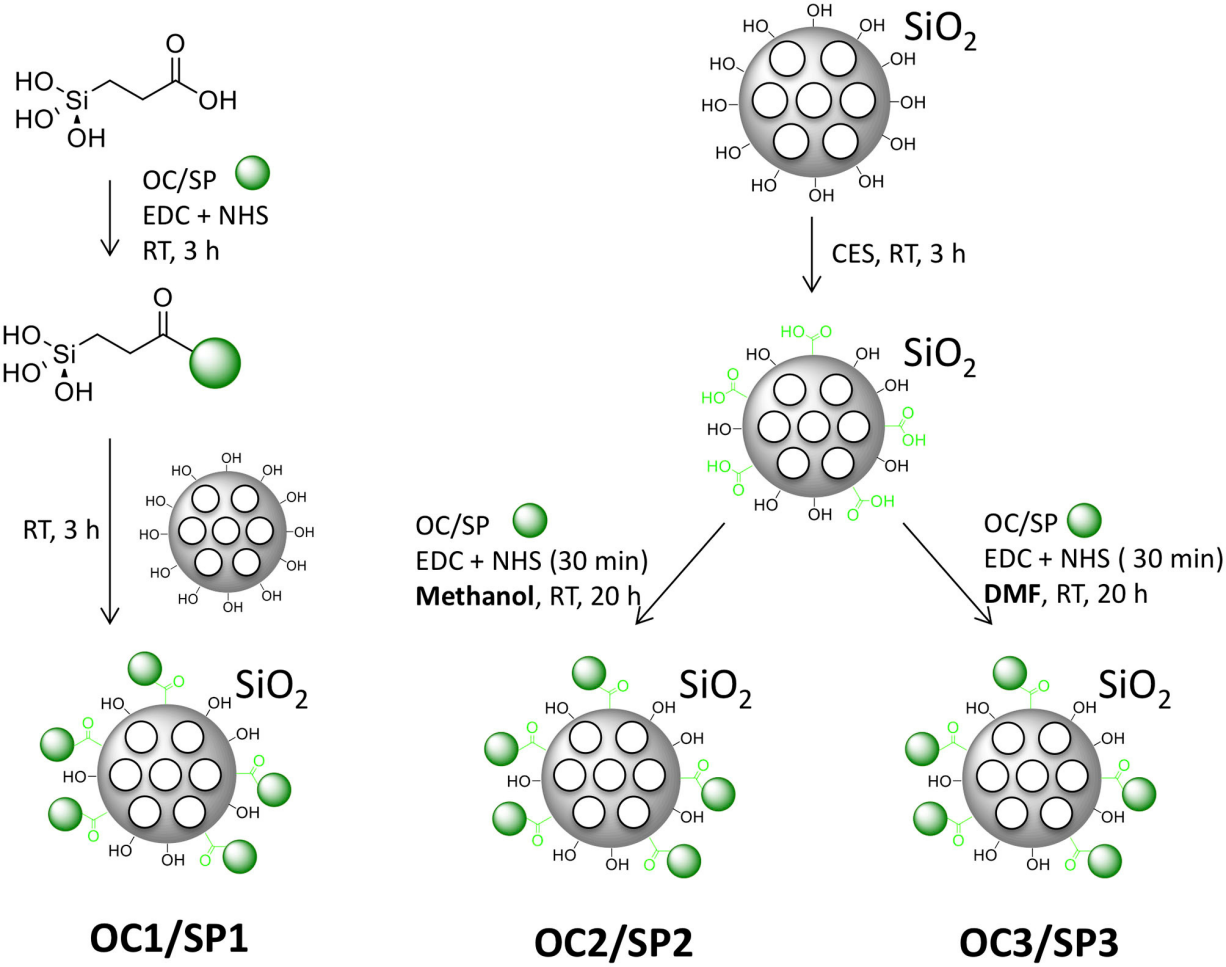
Schematics of the different functionalization strategies for peptide MSN capping, yielding MSN_OC1_, MSN_SP1_, MSN_OC2_, MSN_SP2_, MSN_OC3_, and MSN_SP3_.

Three different means for attaching the protected peptides to the MSNs were evaluated: silane coupling and EDC/NHS coupling from two different solvents. The silane coupling of the peptide to the nanoparticles was performed by first linking carboxyethylsilantriol (CES) to the α-amino acid of the peptides by EDC/NHS-activated coupling, followed by base-catalyzed silanization of the calcined MSNs (Figure 2). The resulting MSNs were named MSN_OC1_ and MSN_SP1_. For the EDC/NHS coupling, the MSNs were first surface-functionalized by covalent attachment of CES, followed by activation of the carboxy-functions by EDC/NHS. Peptide attachment was then performed in either methanol (MSN_OC2_ and MSN_SP2_) or DMF (MSN_OC3_ and MSN_SP3_; Figure 2). Further, the derived MSNs were covalently labeled with the red fluorescent dye (ATTO647N) to make them detectable by optical methods (Supplementary Table 1).

As the final step, the peptides were de-protected by dispersing the peptide-MSN conjugates in 95% trifluoroacetic acid (TFA) for 2 h at RT, followed by two washing steps with diethylether and cyclization of the peptides in 5% acetic acid (pH = 6.0), containing 20% dimethyl sulfoxide (DMSO) for 4 h at RT. For SP, DMSO was replaced by 5% acetic acid in order to prevent cyclization.

### Physicochemical Characterization of Peptide-Functionalized MSNs

**Figure 3:**
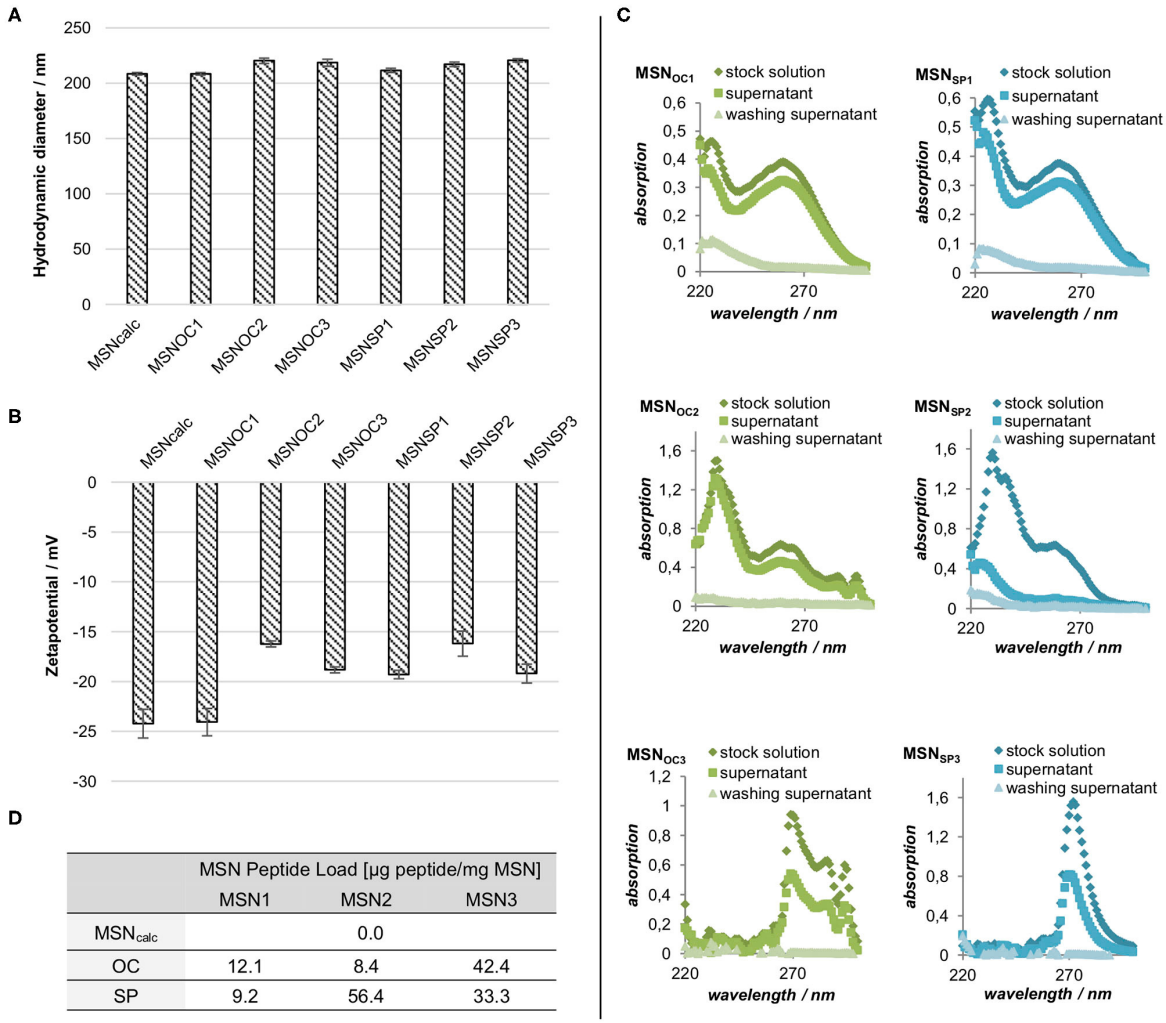
**(A)** Hydrodynamic diameter and **(B)** zeta potentials of differently-functionalized MSNs in 1 mM KCl (pH = 5.0). Averages of three replicate measurements with SD are shown. **(C)** UV/Vis spectra of the peptide stock solutions and the resulting supernatants after peptide conjugation and nanoparticle washing steps for MSN_OC1−3/SP1−3_. **(D)** Peptide load per mg of dry MSN weight. The amount of MSN-bound peptide was estimated from the above measurements **(C)** by subtracting absorption values of the supernatant and the wash from the absorption of the original peptide stock solution with subsequent conversion via calibration curves.

Peptide functionalization had no impact on the dispersibility of the MSNs, as demonstrated by the identical MSN hydrodynamic radii, measured in 1 mM KCl (pH = 5.5; Figure 3A). Apart from MSN_OC1_, all the particles demonstrated a minor reduction of negative zeta potential upon peptide attachment. The carboxy-silane functionalized MSNs and MSN_OC1_ had a zeta-potential of −25 mV, while zeta-potentials of all the other peptide-functionalized MSNs were in the range of −15 to −20 mV (Figure 3B). As all MSNs had comparable negative surface charge, any particle charge-related effects on potential differences between the interactions of different MSNs with cells can thus be assumed to be minor.

The amount of the attached peptides was determined indirectly by UV/Vis spectroscopy of the stock peptide solutions and the derived supernatants after conjugation (Figures 3C,D). The amount of attached peptide was clearly lower for the MSN_OC1_, MSN_SP1_, and MSN_OC2_ as compared to MSN_SP2_, MSN_OC3_, and MSN_SP3_, showing that silane coupling was not very efficient under the studied conditions. Differences between attachment efficiencies of OC and SP for the EDC/NHS couplings are suggested to originate from solubility differences between the peptides. While both peptides show very good solubility in DMF, the solubility of OC in methanol is not as good as that of SP. Solubility effects could also have contributed to the low efficacy of the silane coupling, since peptide functionalization with CES also lowers peptide polarity, in turn affecting peptide solubility in the utilized solvents (water/methanol). An additional influence of partial self-condensation of the peptides cannot be excluded in this case. Of note, the indirect means of measuring the peptide attachment used, i.e., UV/Vis spectroscopy of the reaction supernatants, does not allow distinguishing between the covalent attachment and physisorption of the targeting peptides to MSNs.

### MSNs Do Not Shed Targeting Peptides Upon Reconstitution in an Aqueous Buffer

**Figure 4:**
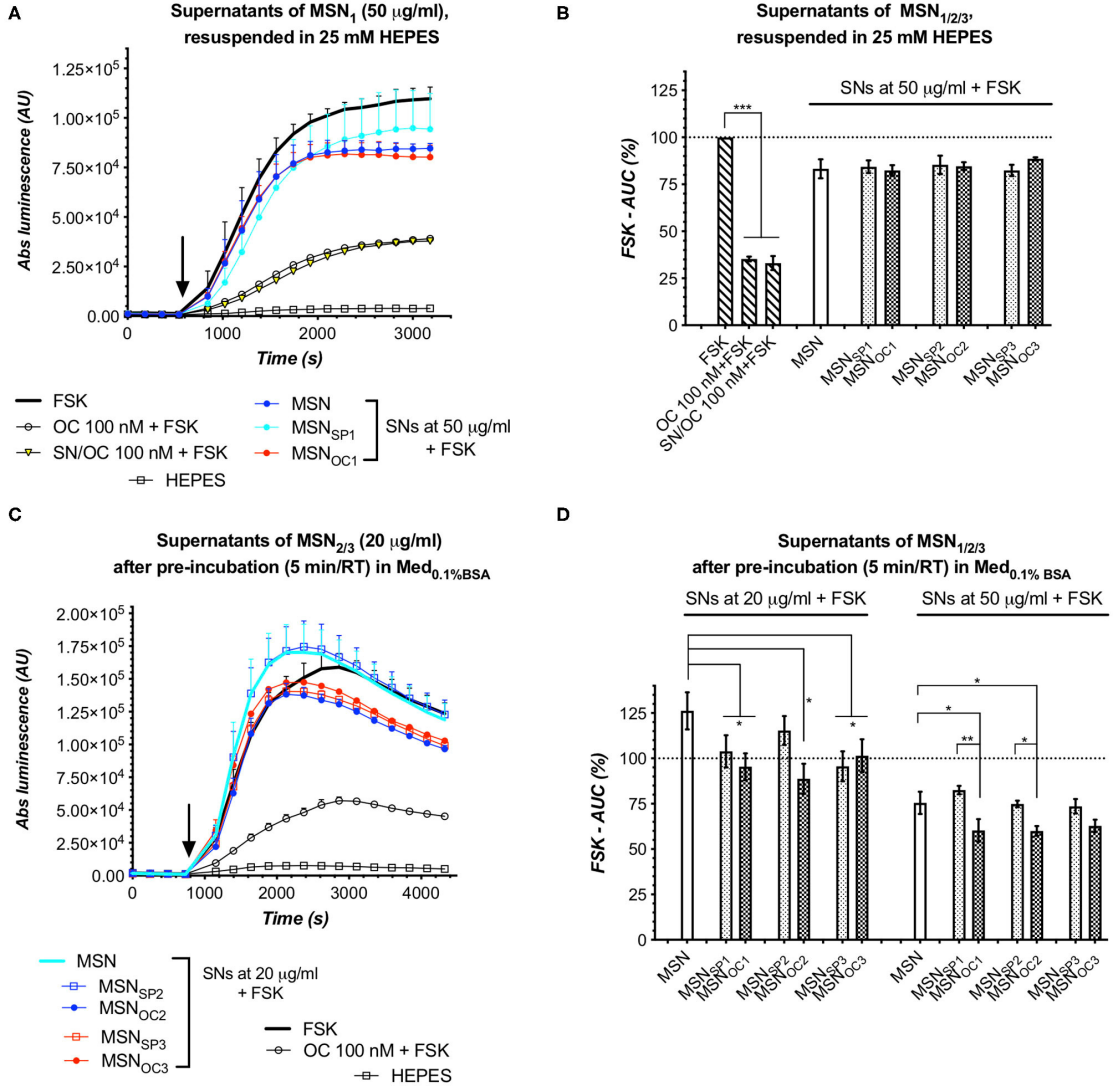
MSN supernatants (SNs) in targetability bioassay with SSTR2 sensor cells. SNs, obtained by centrifugation of either freshly prepared HEPES (25 mM, pH 7.4, at RT) suspensions of MSN_1/2/3_ **(A,B)** or after spiking of the latter to MED_0.1%BSA_ **(C,D)**, were added to HEK-GS/SSTR2_HA sensor cells. Similar luminescent responses to all the studied HEPES SNs (of MSN vs. MSN_SP_ vs. MSN_OC_) confirm that neither of MSN species contained significant amount of free targeting peptides when suspended in HEPES buffer. On the contrary, responses to SNs, collected 5 min after MSN spiking to Med_0.1%BSA_, reveal varying degrees of rapid and MSN dose-proportional targeting peptide shedding, with MSN_OC3_ releasing much less of the peptide as compared to MSN_OC1_ and MSN_OC2_. **(A,C)** depict luminescence reads from single representative experiments with the indicated MSN species. Error bars denote mean ± SD (only SD's upper half is shown); y- and x-axes denote luminescence signal (AU) and time (s), respectively; the moment of MSN/compound addition is indicated with the black arrow. **(B,D)** demonstrate integrated results [by means of FSK-AUC (%) values] of several independent runs. Error bars, average values ± SEM. Statistics, one-way repeated measures ANOVA with Tukey's correction for multiple comparisons; all the comparisons with the significance level <0.05 are indicated with asterisks. All the assays were run at standard conditions, with IndMed_0.1%BSA_. Each run in 3× technical replicates; the number of individual assay repeats (n#) for bar diagrams ≥3.

Assuming that free and MSN-bound OC shares the same affinity to SSTRs, our bioassay for targetability would not necessarily be able to discriminate between these peptide species, which could give rise to false-positive results if MSN prep has a significant amount of the liberated peptide admixed. Thus, in order to validate targetability of a nanoformulation, a possible input from contaminating free (non-bound to nanoparticles) peptides has to be excluded (Paramonov et al., 2018). To this end, we harvested the supernatants (SNs) of MSNs immediately after their reconstitution (from lyophilized stocks) in aqueous HEPES buffer (25 mM, pH 7.4, at RT) and subjected them to the targetability bioassay with HEK-GS/SSTR2_HA cells. Here, SNs of all the MSN subtypes exerted similar response, with SNs of MSN_OC1−3_ yielding luminescent curves indistinguishable from those of MSN and MSN_SP1−3_ at the same dose, which virtually virtually excludes the presence of measurable amounts of free OC [the sensitivity threshold for free OC is *ca* 1 nM in SSTR2 bioassay (Paramonov et al., 2018)] in the freshly prepared aqueous MSN suspensions (Figures 4A,B).

Noteworthily, the SNs of all the MSNs in question appeared to have a minor inhibitory effect on intracellular cAMP levels in the sensor cells as revealed through comparison to FSK response. This effect apparently cannot be attributed to free OC/SP, for the SN of non-capped MSN (*a fortiori* devoid of any targeting peptides) evoked the same response. However, this effect, though minor, was highly reproducible and could be speculatively attributed to some dissolution products of MSNs, which could affect the sensor cells and/or their responsiveness to FSK (refer also to Supplementary Figure 6). Such a high responsiveness of sensor cells to adulterating compounds, including trace amounts of organic solvents, was noted earlier (Paramonov et al., 2018). This specifically underlines the importance of the matched parallel assay design, i.e., when MSN_OC_ are compared to MSN and MSN_SP_ in the same assay and in the same dose, thus allowing control for possible confounding effects and their possible non-linearity, eventually ensuring the validity of the results.

### MSNs Start Shedding Targeting Peptides Upon Entry to Culture Medium With 0.1%BSA

MSN_SP/OC1_, MSN_SP/OC2_, and MSN_SP/OC3_ were firstly evaluated for targetability under simplistic conditions, i.e., by adding freshly prepared MSN suspensions in aqueous HEPES buffer directly to the sensor cells, cultured in a medium with 0.1% BSA (Med_0.1%BSA_). To study MSNs for possible peptide detachment in the BSA-containing medium, we added HEPES-suspended MSNs to Med_0.1%BSA_, collected SNs after 5 min incubation at RT, and subjected the SNs to the targetability assay.

**Figure 5:**
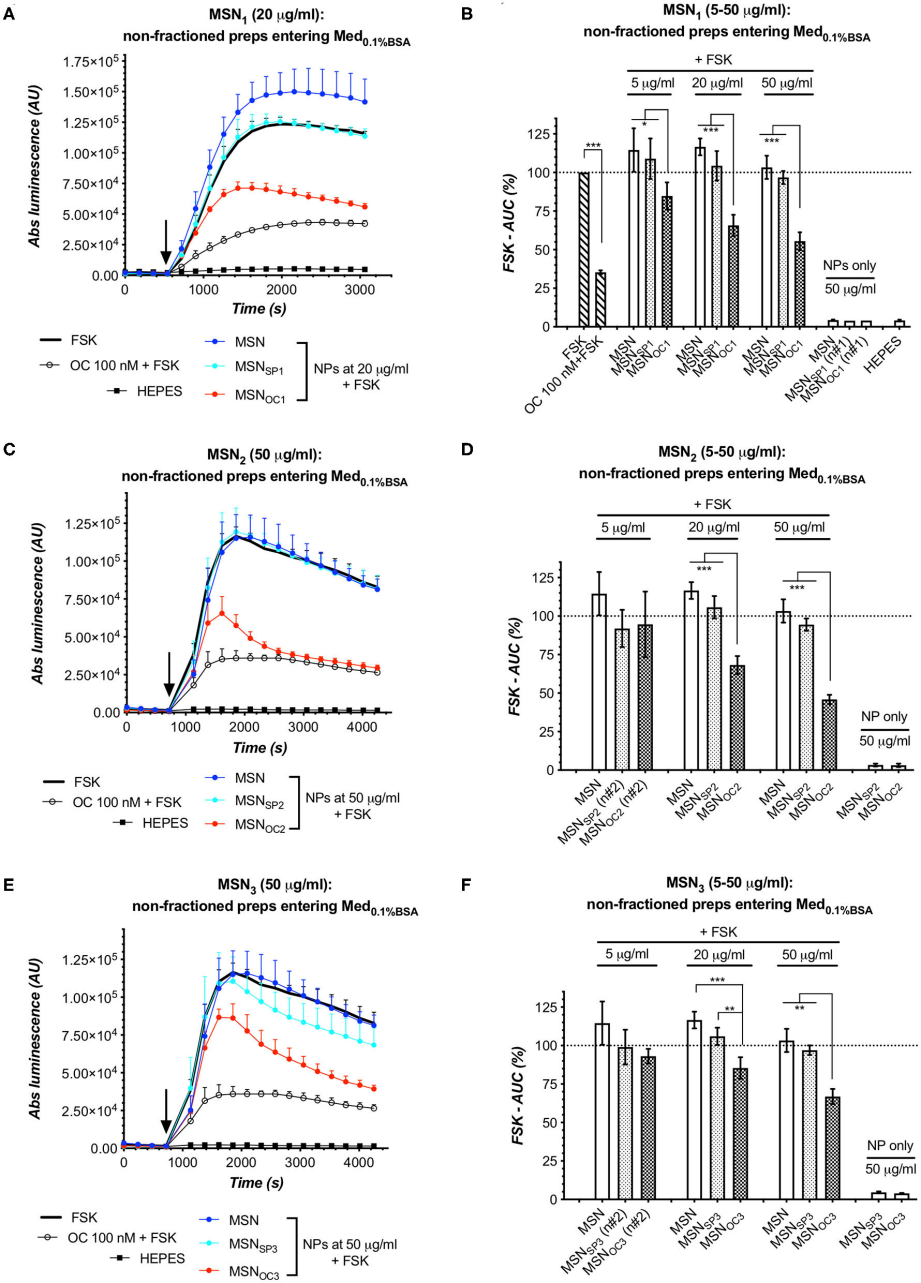
MSN_OC1_ and MSN_OC3_ activate SSTR2 in the medium with 0.1% BSA. Evoked responses in the sensor cells (HEK-GS/SSTR2_HA) upon exposure to non-fractionated MSNs (suspended in HEPES) in IndMed_0.1%BSA_. As SN studies of MSN_1_ and MSN_3_ in HEPES and Med_0.1%BSA_ (Figures 4A–D, respectively) demonstrate that neither of these MSNs shed significant amounts of targeting peptides (at doses of ≤20 and ≤50 μg/ml for MSN_OC1_ and MSN_OC3_), the observed cAMP responses for MSN_OC1_ at 5–20 μg/ml and MSN_OC3_ at 20–50 μg/ml could only be attributed to SSTR2 activation by OC on the surface of nanoparticles, confirming their targetability under the studied conditions. **(A–F)** Luminescence reads in the sensor cells exposed to non-fractionated MSN_1_, MSN_2_, and MSN_3_; data from single representative experiments and integrated results of several independent runs [panels **(A,C,E)** and **(B,D,F)**, respectively]. All the assays were run at standard conditions, with IndMed_0.1%BSA_. Each run in 3× technical replicates; the number of individual assay repeats (n#) for bar diagrams ≥3 if not indicated otherwise. *For luminescence curves* **(A,C,E)**: error bars denote mean ± SD, with only SD's upper half shown. y- and x-axes denote luminescence signal (AU) and time (s), respectively; the moment of MSN/compound addition (spiking) is indicated with the black arrow. *For bar diagrams* **(B,D,F)**: y-axis depicts %FSK-AUC values (response to FSK taken for 100%), derived from the luminescence signals. Error bars represent average values ± SEM. Statistics: one-way repeated-measures ANOVA with Tukey's correction for multiple comparisons; all the comparisons with the significance level below <0.05 are indicated with asterisks (further information in Materials and Methods section).

Interestingly, all the OC-capped MSNs released at least some quantity of the targeting peptide into the liquid phase upon exposure to Med_0.1%BSA_, with the amount of the liberated OC being proportional to the MSN dose and varying between MSN species (Figures 5C,D). Specifically, MSN_OC2_ shed enough of OC to activate SSTR2 at 20–50 μg/ml, which is revealed through comparisons with the matched SNs of non-capped MSN and MSN_SP2_. MSN_OC1_ released less of OC under the same conditions, with significant SSTR2 activation by SN of MSN_OC1_ only observed at 50 μg/ml. MSN_OC3_ clearly demonstrated the least propensity for peptide shedding: at 20 μg/ml, SNs of MSN_OC3_ and MSN_SP3_ evoked the same response; at 50 μg/ml, SN of MSN_OC3_ appeared to specifically inhibit cAMP, but the comparison with the respective SN of MSN_SP3_ did not reach significance.

These results are in line with a recent study showing that the release of a viral peptide from MSNs was much faster in the presence of serum proteins as compared to the protein-free buffer. The competitive adsorption of serum proteins to the MSNs leading to detachment of the pre-adsorbed peptide was suggested to account for this observation (Braun et al., 2020). Thus, together with the discussed solvent-dependent differences in targeting peptide solubility during MSN peptide functionalization step, and the related differences in the extent of peptide physisorption, this evidence highlights the importance of pre-evaluating the peptide conjugation stability under complex conditions (presence of proteins).

### MSN_OC1_ and MSN_OC3_ Demonstrate Targetability in the Medium With 0.1%BSA

Next, we studied the non-fractionated MSNs preps for targetability in Med_0.1%BSA_. Here, MSN_OC2_ and MSN_OC3_ clearly activated SSTR2 as compared to the matched doses of respective non-capped MSN and MSN_SP_, with the effect becoming significant at 20 μg/ml of MSNs and rising further with dose. MSN_OC1_ dose-dependently activated SSTR2 already from 5 μg/ml onwards (Figures 5A–F, respectively). MSN_SP1_, MSN_SP2_, and MSN_SP3_ did not significantly differ from the non-capped MSNs in terms of the evoked response, which excludes significant SSTR activation with these treatments (Figures 5B,D,F).

When aligned with the reviewed SN data, it becomes evident that that MSN_OC1_ and MSN_OC3_ are targetability-competent in Med_0.1%BSA_, as these MSNs species did not release significant amounts of OC into the liquid phase when suspended in either HEPES buffer (at doses ≤50 μg/ml; Figure 4B) or Med_0.1%BSA_ (at doses ≤20 μg/ml and up to at least 50 μg/ml, respectively; Figure 4D), while retaining SSTR-activating potential. Collectively, this evidence attributes the observed cAMP inhibitory effects of MSN_OC1_ and MSN_OC3_ (at doses of 5–20 and 20–50 μg/ml, respectively) to MSN-bound OC, thus validating their targetability under the studied conditions.

MSN_OC2_ shed significant amounts of free OC in Med_0.1%BSA_ already at 20 μg/ml, which was also the lowest dose, triggering significant SSTR activation with non-fractionated MSN_OC2_ (Figure 5D). With this, the deduction of the input of MSN-anchored OC into the net SSTR activation is problematic, precluding the targetability claim for MSN_OC2_ in Med_0.1%BSA_.

### MSN_OC3_ Retain Targetability in the Presence of Serum Proteins

Finally, to study the effects of serum proteins on MSN targetability, we resorted to the corona formation setup, mimicking the protein build-up on MSN surface after their retention in systemic circulation *in vivo*. MSN_OC3/SP3_ were selected for corona experiments, for these nanoparticles demonstrated the least propensity for peptide shedding in Med_0.1%BSA_. To allow for corona formation on nanoparticles, fresh MSN suspensions (5 mg/ml; non-capped MSN vs. MSN_OC3_ vs. MSN_SP3_) in HEPES were transferred to Med_10%iFBS_ (yielding 1.5 mg/ml of MSNs at 7% w/v iFBS) and incubated for 60 min at +37°C with agitation, before being subjected to analysis.

SDS-PAGE analysis of the acquired coronas revealed that MSN_OC3_ and MSN_SP3_ had similar corona compositions, with both nanoparticle species adsorbing less protein as compared to non-capped MSNs (Supplementary Figure 5). Before proceeding with MSN targetability validation in the presence of corona, we ensured that (1) during the corona build-up, i.e., 1-h incubation at +37°C in serum-supplemented medium, free OC retains stability and signaling competence, and that (2) MSN_OC3_ do not shed significant amounts of targeting peptides under the same conditions (1 h and +37°C) once suspended in aqueous HEPES buffer (Supplementary Figures 6A–C, respectively). With this, we excluded the possibility of OC degradation during the pre-incubation phase as a possible cause of false-negative results and eliminated temperature and time factors as driving forces for MSN disintegration.

**Figure 6:**
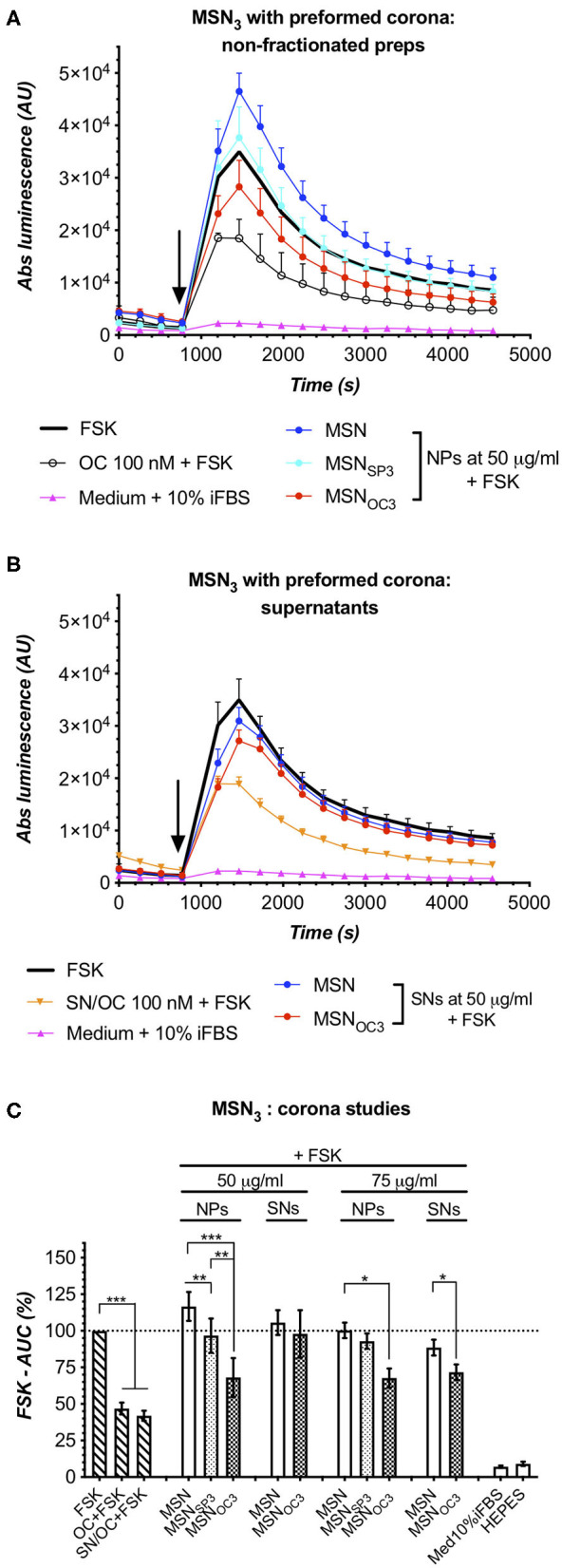
Matched-dose parallel analyses of MSN_3_ and the derived SNs, harvested after incubation in Med_10%iFBS_ for 1h/+37°C, confirm targetability of MSN_OC3_ in the presence of protein corona. Further information in the main text. **(A,B)** Luminescence responses in HEK-GS/SSTR2_HA cells to non-fractionated MSN_OC3_ and the matched SNs, harvested after MSN incubation for 1 h at +37°C in Med_10%iFBS_; data from single representative experiments. Error bars denote mean ± SD, with only SD's upper half shown. y- and x-axes denote luminescence signal (AU) and time (s); the moment of MSN/compound addition is indicated with the black arrow. **(C)** Integrated results of several independent runs for MSN/MSN_SP3_/MSN_OC3_ and the matched SNs under the corona setup. y-axis, luminescence-derived %FSK-AUC values (FSK response taken for 100%); error bars, average values ± SEM. Inferential statistics, either repeated-measures ANOVA with mixed-effects model and Tukey's multiple comparisons test or paired one-tailed *t*-test (for number of groups under comparison ≥3 or 2, respectively). All the comparisons with the significance level below 0.05 are indicated with asterisks; more information in the Materials and Methods section. The assays were run at standard conditions, with IndMed_10%iFBS_. Each run in 3× technical replicates; bar diagrams encompass data from ≥3 individual repeats.

MSN targetability studies in IndMed_10%iFBS_ turned out to be somewhat technically more challenging as compared to assays in Med_0.1%BSA_, for serum clearly affected the performance of the sensor cells, narrowing the dynamic range [best perceived through comparison of FSK-normalized (100%) responses to 100 nM free OC in Med_0.1%BSA_ and Med_10%iFBS_, estimated as *ca* 35 and 47%; Figures 5B, 6C] and increasing noise level in the assay. Despite these difficulties, partially ameliorated by increasing MSN dose, targetability studies of MSNs after corona formation yielded unambiguous results: MSN_OC3_ specifically activated SSTR2 in the sensor cells at 50 μg/ml, which in the absence of significant free OC shedding, verified by probing the matched SNs, directly confirmed the retained ability of MSN_OC3_ to engage the targeted receptors even in the presence of corona (Figures 6A–C). Targetability validation at higher doses of MSN_OC3_ (75 μg/ml) was not possible due to significant OC liberation by nanoparticles (Figure 6C).

## Discussion

The main objectives of the present work were to develop an actively targeted nanocarrier and to validate its targetability under biologically relevant conditions *in vitro*, thus fulfilling the prerequisites for subsequent *in vivo* translation. To this, we functionalized MSNs with short peptide ligands to SSTRs via differential linking chemistries and studied the resulting nanocarriers for targetability in a sensitive bioassay that measures receptor activation in the membranes of living cells with the ligands on nanoparticles. We evaluated MSNs both under protein-depleted and serum-enriched conditions, confirming MSN targetability even in the presence of protein corona. Our work not only illuminates biologic events, surrounding targetability, but also provides important insights into the applied functionalization strategies. We expect that the experimental conclusions with 180 nm MSNs, utilized in this study, could also be broadly applied to MSNs of similar design and size between 100 and 200 nm, assuming negligible curvature effects across this size range.

In terms of the linking chemistry, it is important to note that even if no clear spacer was used for the peptide attachment, active targeting with MSN_OC1_ and MSN_OC3_ could still be clearly demonstrated. Targetability validation in case of MSN_OC2_ was hindered due to excessive peptide shedding upon nanoparticle entry to culture medium.

Studies with MSN_OC1_ and MSN_OC3_ in Med_0.1%BSA_ (non-corona setup) also revealed intriguing discrepancy between the net amount of MSN-associated peptides and MSN capacity to activate targeted receptors. MSN_OC1_ clearly activated SSTR2 already at 5 μg/ml, with the effect increasing further with dose, whilst MSN_OC3_ was only able to activate the receptors from doses ≥20 μg/m (Figures 5B,F). In other words, despite being less abundant (MSN_OC1_ carries *ca* 3.5-fold less of OC attached as compared to MSN_OC3_; Figure 3C), peptides on MSN_OC1_ were able to activate SSTR2 more potently. Admittedly, proper understanding of the relation between MSN ligand load and the evoked receptor response is of high practical significance, which warrants further research. Phenomena, possibly emerging upon ligand presentation from a surface at nanoscale, such as targeting peptide crowding with ensuing steric hindrance or differential propensity to activate receptors, as well as targeting peptide effects on MSN opsonization, altering dynamics of MSN physical interaction with plasma membranes of the sensor cells, are among the possible underlying mechanisms of the observed response.

In the present work we paid special attention to the negative controls for MSN targetability validation. Indeed, in view of the amassed evidence, highlighting the functional surface as a principal determinant of nanocarrier performance in living systems (Albanese et al., 2012; Walkey et al., 2012; Stylianopoulos and Jain, 2015), it is surprising how often scrambled moiety-decorated nanoparticles are being omitted from the control set, thus undermining the robustness of the obtained experimental data. Herein, we designed and validated a scrambled peptide, which is structurally close to the active targeting ligand OC, but has no specific affinity for SSTR2, 3, and 5. SP-decorated MSNs did not exert specific effects on intracellular cAMP in the sensor cells either in medium with 0.1%BSA or under preformed corona conditions, with the exclusion of a single case (MSN_SP3_ decreased cAMP as compared to MSN at 50 μg/ml after corona formation; Figures 6A,C), which qualifies MSN_SP_ as a proper negative control under the present targetability setup. However, in view of the poor predictability of the effects of peptide functionalization on MSN performance in complex biotic media, it is in the studies of targetability-associated downstream events, e.g., MSN cellular uptake, where MSN_SP_ would be essential to determine the actual influence of specific receptor engagement on the net MSN internalization rate.

We also want to stress the importance of the identified phenomenon of targeting ligand shedding that takes place upon the passage of MSN from an aqueous solution to a more complex medium. Accounting for such a phenomenon is absolutely instrumental for adequate interpretation of a bioassay's readout. From a nanoparticle characterization/functionalization point of view, our results also highlight the importance of evaluating peptide attachment stabilities under biologically relevant conditions.

Targetability studies of MSNs with the preformed corona yielded, perhaps, the most relevant practical evidence. Indeed, opsonization with biologic macromolecules is a crucial part of “the life cycle” of any nanocarrier in living systems, with the processes of corona build-up starting immediately upon administration and continuously evolving along with the changes of microenvironment, dynamically altering the outer interphase of nanoparticles and affecting their interactions with the living matter (Caracciolo et al., 2017; Cai and Chen, 2019; Francia et al., 2019). We successfully validated the ability of MSN_OC3_ to specifically engage the targeted receptors even in the presence of serum proteins—a situation mimicking the expected scenario for MSNs under systemic administration.

Taken together, our work highlights the importance of a holistic approach to targetability validation, where the integrity and functional competence of a nanocarrier have to be confirmed at all the stages *en route* to the targeted lesion. By taking the alleged stability of a nanoformulation for granted or by assuming targetability based on late or indirect phenomena, well downstream of and not necessarily related to the immediate targeting ligand-targeted receptor interaction, e.g., uptake of nanoparticles or nanoparticle-related toxicity, it is quite likely to end up with faulty conclusions on targetability and the effects ascribed to it. Unfortunately, such approaches to characterization of targeted nanocarriers are still in common use, which adds to the confusion on the perceived utility of the active targeting concept and hinders further progress in the field. To this end, we also want to specifically stress the importance of *in vitro* characterization of nanocarriers. Indeed, as demonstrated in the present work, with a tailored experimental setup, one can address such intricate processes as corona formation and targeted receptor engagement. These and the related phenomena are very difficult to study *in vivo* at good resolution presently, so proper scrutiny *in vitro* stands out as a virtually indispensable step, safeguarding against the entry of under-characterized (and thus conceivably non-functional) nanoformulations into *in vivo* studies. We believe our study represents a robust example of *in vitro* targetability validation of a nanocarrier and as such would be of further utility not only for projects on GPCR targeting but also for the incentives on active targeting with nanoparticles in general, highlighting the inherent difficulties with targetability validation and aiding in experimental design.

## Data Availability Statement

The raw data supporting the conclusions of this article will be made available by the authors, without undue reservation.

## Author Contributions

CS, AR-M, VP, and ML conceptualized the overall project. AR-M and VP conceived and established the targetability bioassay. ML and MG developed the chemical design. MG undertook synthesis and physicochemical characterization of nanoparticles. VP carried out targetability studies with nanoparticles, as well as free peptide receptor engagement and toxicity studies with living cells. CS, AR-M, and ML supervised the research and data analysis. VP, MG, and ML drafted the manuscript. All the authors critically read, amended, and approved the final version of the manuscript.

## Conflict of Interest

The authors declare that the research was conducted in the absence of any commercial or financial relationships that could be construed as a potential conflict of interest.

## Abbreviations

ATCC: American type culture collection
AU: arbitrary units
AUC: area under curve
cAMP: 3′-5′-cyclic adenosine monophosphate
BET: Brunauer-Emmet-Teller theory
BSA: bovine serum albumin
CI: confidence interval
CCK-8: cell counting kit-8 (*in vitro* assay for cell viability)
DMEM: Dulbecco's modified Eagle's medium
DMF: dimethylformamide
EDC: N-(3-(dimethylamino)propyl)-N′-ethylcarbodiimide
IndMed: inducing medium
FSK: forskolin
GPCR/GPCRs: G protein-coupled receptor/s
HEK-GS: HEK293 cell line with stable overexpression of GloSensor-22F cAMP probe
IC50: concentration of a compound triggering half of the maximum inhibitory effect
iFBS: heat-inactivated fetal bovine serum
MALDI-TOF MS: matrix-assisted laser desorption ionization – time of flight mass spectrometry
MSN/MSNs: mesoporous silica nanoparticle/s
NHS: N-hydroxysuccinimide
NLDFT: non-local density functional theory
ns: non – significant
OC: octreotide
ON: overnight
RT: room temperature
SDS-PAGE: sodium dodecyl sulfate-polyacrylamide gel electrophoresis
SD: standard deviation
SEM: standard error of the mean
SN/SNs: supernatant/s
SP: scrambled peptide
Sst14: somatostatin-14
SSTR/SSTRs: somatostatin receptor/s
TEM: transmission electron microscopy
TFA: trifluoroacetic acid
WB: western blotting.
