# Supplementary material for: In vitro Targetability Validation of Peptide-Functionalized Mesoporous Silica Nanoparticles in the Presence of Serum Proteins
Source: Front Chem. 2020 Nov 13;8:603616. doi: 10.3389/fchem.2020.603616 (PMC7691633; doi:10.3389/fchem.2020.603616)
Supplement: Supplementary file 1 [file Table_1.docx]

Supplementary Material

Table S1. Relative fluorescence of the ATTO647N-labeled MSNs at 680 nm (fluorescence of calcinated MSNs taken for 1.0; λ_exc_ = 635 nm). As the anchoring groups for ATTO647N are present both inside and outside of the mesopore system of the nanoparticles, it`s expected that the dye moieties have covalently attached within both of the specified compartments. The fraction of dye present inside *vs* outside the mesopore system could not be determined quantitatively due to the absence of proper analytical means.

|  | MSN_calc_ | MSN_OC1_ | MSN_OC2_ | MSN_OC3_ | MSN_SP1_ | MSN_SP2_ | MSN_SP3_ |
| --- | --- | --- | --- | --- | --- | --- | --- |
| Rel. Flour. | 1.0 | 1.2 | 0.9 | 1.2 | 0.5 | 0.8 | 0.6 |

*
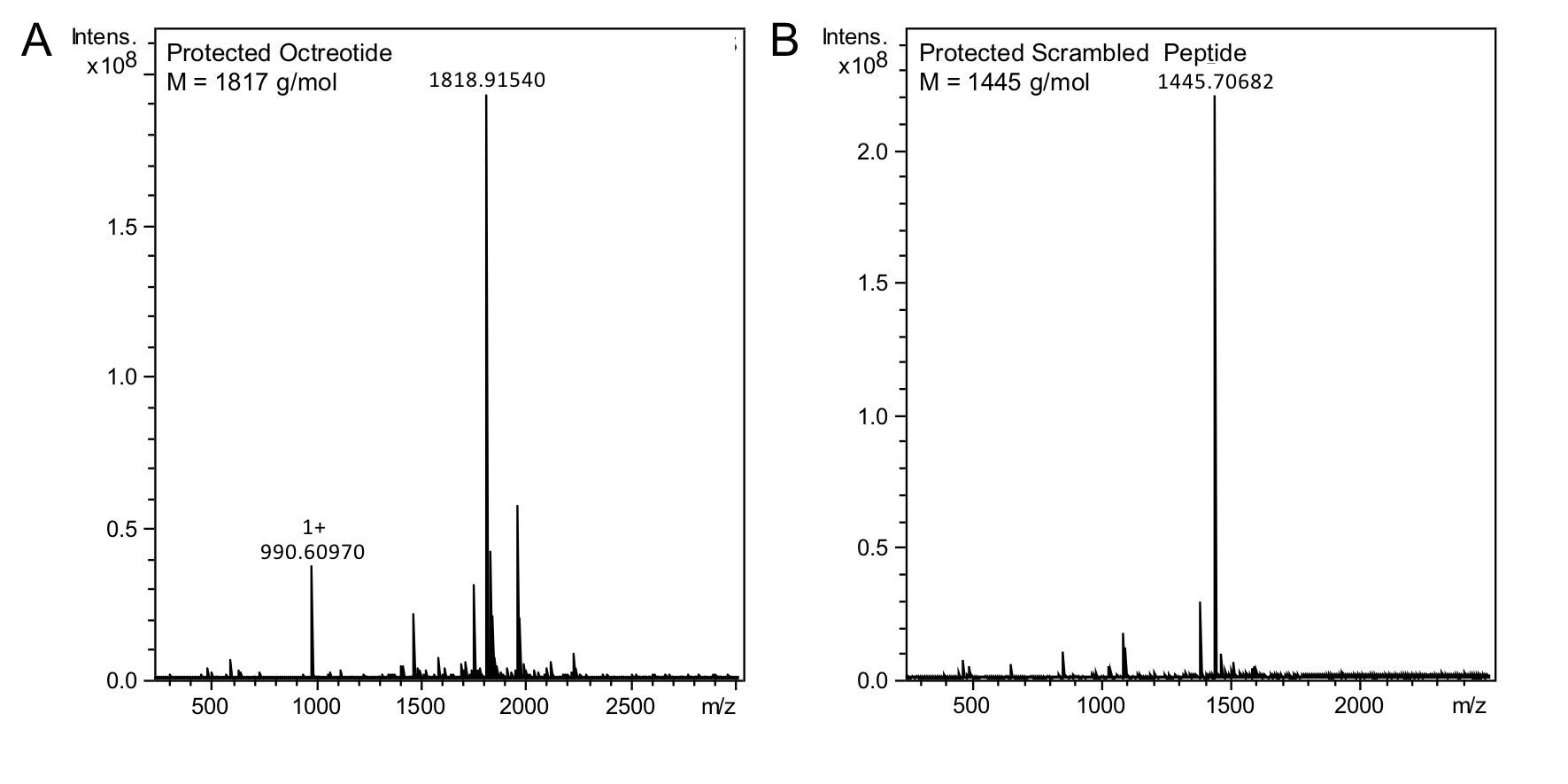
*

Figure S1. MALDI-TOF MS of the side chain-protected octreotide (A) and scrambled peptide (B) after cleavage from the resin with 1% TFA.


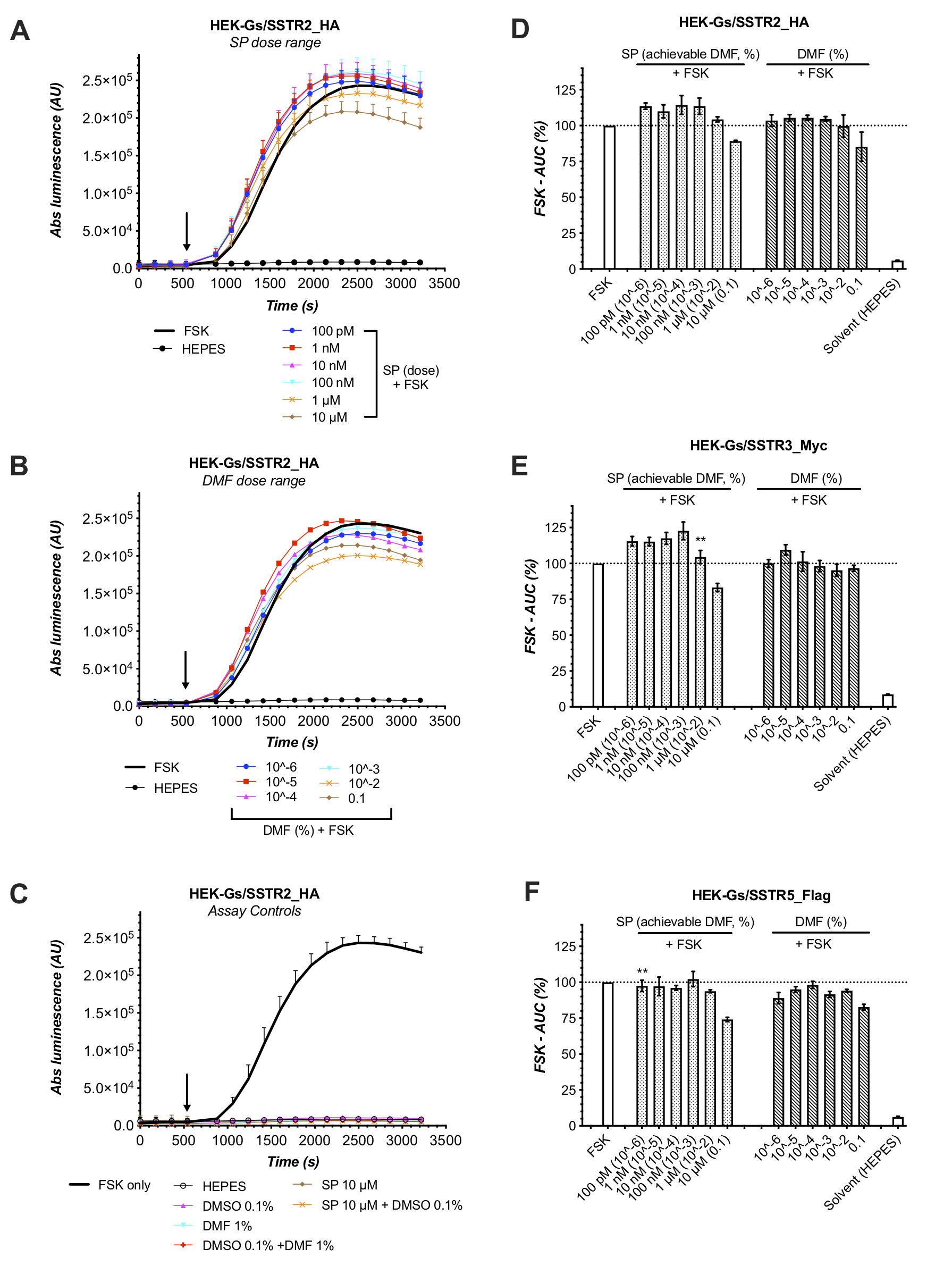


**Figure S2**. Scrambled peptide does not activate SSTR2, 3 or 5 across dose range of 100 pM – 10 μM.

(**A-C**) Luminescence signal (kinetic reads) in HEK-GS/SSTR2_HA cells upon exposure to a range of concentrations of de-protected SP and matching levels of DMF, along with the assay controls (panels A, B and C respectively), in a representative experiment (in 3x technical replicates; error bars denote mean +/- SD, with only SD`s upper half shown; panel B – error bars omitted for visual clarity). After capturing the baseline signal, the compounds were added to the cells (the moment of spiking is indicated with the black arrow) and the luminescence read was continued. y and x – axes denote absolute luminescence (AU) and time (s), respectively. (**D-F**) Dose-response studies of SP and matched levels of DMF in HEK-GS with stable overexpression of SSTR2, 3 and 5 (panels D, E and F respectively). The charts represent three (3x) independent experiments combined (each – in 3x technical replicates), with error bars showing mean +/- SEM. y-axis represents FSK-normalized AUC values (FSK-AUC, %) for the luminescence signal; x-axis denotes the treatments used. Comparisons of responses to matched doses of SP and DMF were carried out with two-tailed ratio t test; significant differences are indicated with asterisks (more info in Materials&Methods section). The assays were run at standard conditions with IndMed_0.1%BSA_. All the working solutions of reagents were prepared in HEPES (25 mM, pH 7.4); SP was originally dissolved in DMF (stock of 10 mM).

Responses to SP and matched levels of DMF did not differ in SSTR2-, 3- or 5-overexpressing cells, which allows to exclude strong agonistic activity of SP towards these receptor types. And though comparisons of signals at 1 μM of SP in SSTR3 cells and at 100 pM of SP in SSTR5 cells with the corresponding DMF levels reached statistical significance (**Figures S2E** and **S2F**, respectively) the absolute differences were very small. DMF was not entirely inert in terms of effects on FSK response, which further justifies the selected dose-matched experimental design, keeping solvent effects in check.


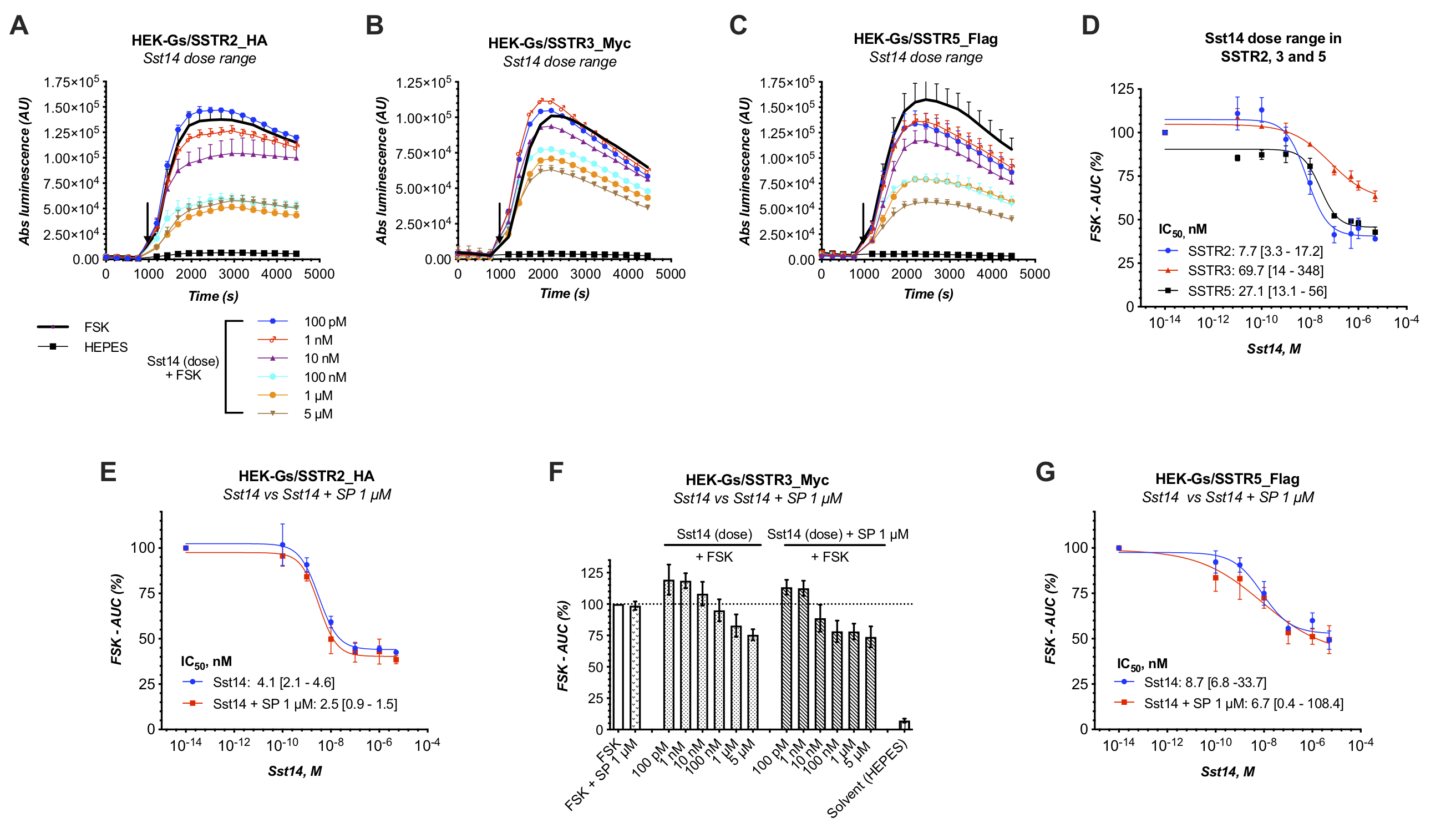


**Figure S3**. Scrambled peptide does not bind to SSTR2, 3 or 5: competition assays with Sst14.

(**A-C**) Luminescent signals in HEK-GS/SSTR2_HA, SSTR3_Myc and SSTR5_Flag cells in response to varying doses of Sst14, captured in single representative experiments (in 3x technical replicates; error bars denote mean +/- SD, with only SD`s upper half shown; panel B – error bars omitted for visual clarity). y and x – axes denote absolute luminescence (AU) and time (s), respectively. The moment of compound addition is indicated with the black arrow. (**D**) Sst14 dose-response curves in HEK-GS/SSTR2_HA, SSTR3_Myc and SSTR5_Flag cells. The curves reflect the combined data from three (3x) independent experiments, with error bars showing mean +/- SEM. y-axis represents FSK-AUC values (%) for the luminescence signal; x-axis denotes Sst14 dose (M). The estimated Sst14 IC_50_ values [with 95% CI, nM] for SSTR2, 3 and 5 are shown. (**E-G**) Competition studies in HEK-GS with overexpression of SSTR2, 3 and 5: Sst14 (dose range) *vs* Sst14 (dose range) plus fixed concentration (1 μM) of de-protected SP. Raw kinetic luminescence signals were rendered into FSK-normalized AUC values (response to FSK taken for 100%) and the latter were either plotted as a bar chart (panel F for SSTR3; bars are shown instead of the derived curves due to the poor global fit) or used for dose-response curve fitting and IC_50_ [with 95% CI, nM] estimation (panels E and G for SSTR2 and 5 respectively), as described in Materials&Methods section. Integrated results of three (3x) independent experiments (each – in 3x technical replicates) are presented; error bars depict mean +/- SEM. To account for the possible effects of DMF in SP-treated samples (SP stock of 10 mM in DMF was used for working solutions preparation, which corresponds to DMF concentration of 0.01% at 1 μM of SP), all matching samples received identical doses of DMF. The assays were run at standard conditions, with IndMed_0.1%BSA_.

Sst14 dose range studies reveal that excess of SP (1 μM) has no significant effect on cAMP response to Sst14 in HEK cells overexpressing SSTR2, 3 or 5, which efficiently excludes high affinity binding of SP to these receptor subtypes. The estimated IC_50_ values for Sst14 *vs* Sst14 + SP (1 μM) were not significantly different for SSTR2 and 5 (extra sum-of-squares F test, p ≤0.05; panels E and G). And though curve fitting with ensuing IC_50_ comparison for SSTR3 were not feasible due to the overall poor fit, addition of 1 μM of SP did not exert any clear effects on the magnitude and pattern of Sst14 response in HEK-GS/SSTR3_Myc cells across the dose range studied (panel F). Together with the lack of agonistic activity (**Figure S2**), this data confirms that SP is not an efficient binder of SSTR2, 3 or 5 across low nM – low μM dose range and thus qualifies for a control peptide in subsequent experiments.

**
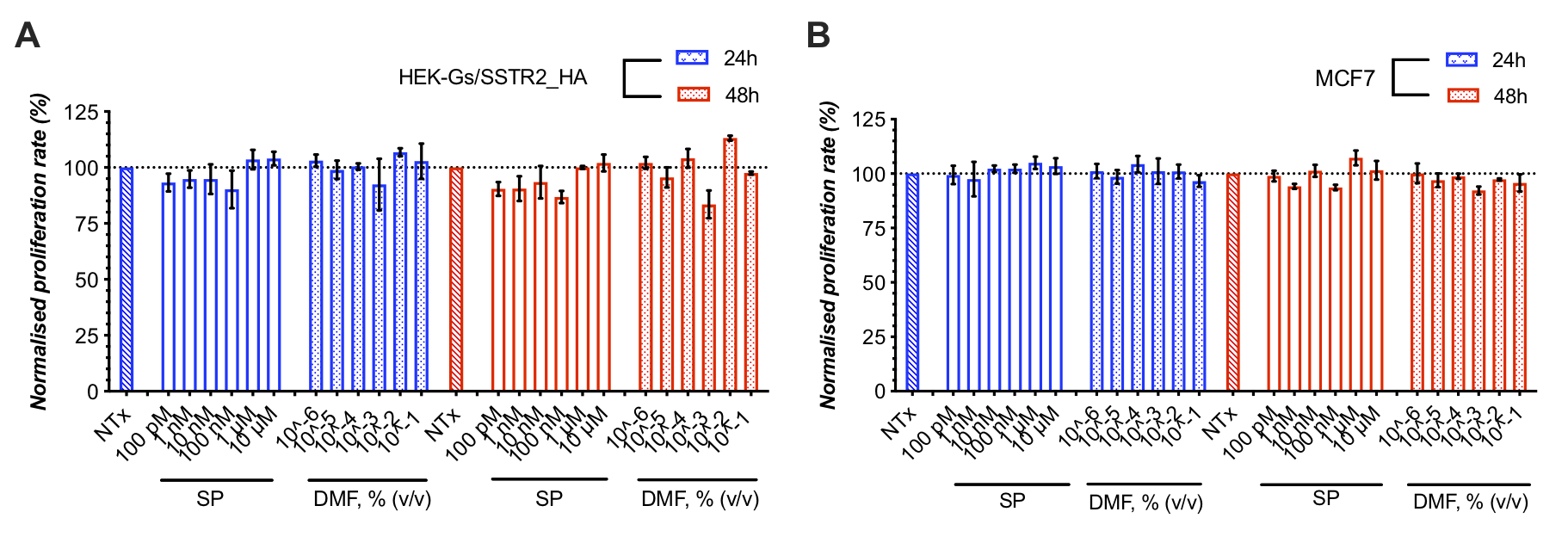
**

**Figure S4**. Scrambled peptide does not affect *in vitro* viability of two unrelated cell lines with differential SSTR2 expression.

(**A**/**B**) CCK-8 assay with SP (100 pm-10 μM for 24 and 48h) in HEK-GS/SSTR2_HA or MCF7 cells, respectively. As the stock solutions of de-protected SP were in DMF (10 mM), the matched levels of DMF (%, v/v), achievable with the indicated SP doses, were included in the runs as well. Proliferation rate was normalized to the corresponding value of non-treated cells (NTx, taken for 100%) that received just HEPES solution. Triton X-100 at 1% (v/v) final concentration was used for positive control – the resulting Abs@450 nm values equaled the ones of the blanks (empty medium), indicating total cell death (bars not shown). The estimated protein abundance of SSTR2, which is the principal target of the active targeting peptide OC, in HEK-GS/SSTR2_HA is *ca* 2-fold of the one of MCF7 cells (Paramonov et al., 2018). The assay was performed 3x and 2x times for 24 and 48h exposures, respectively (each sample – in technical triplicates); the bars represent average values +/- SEM.


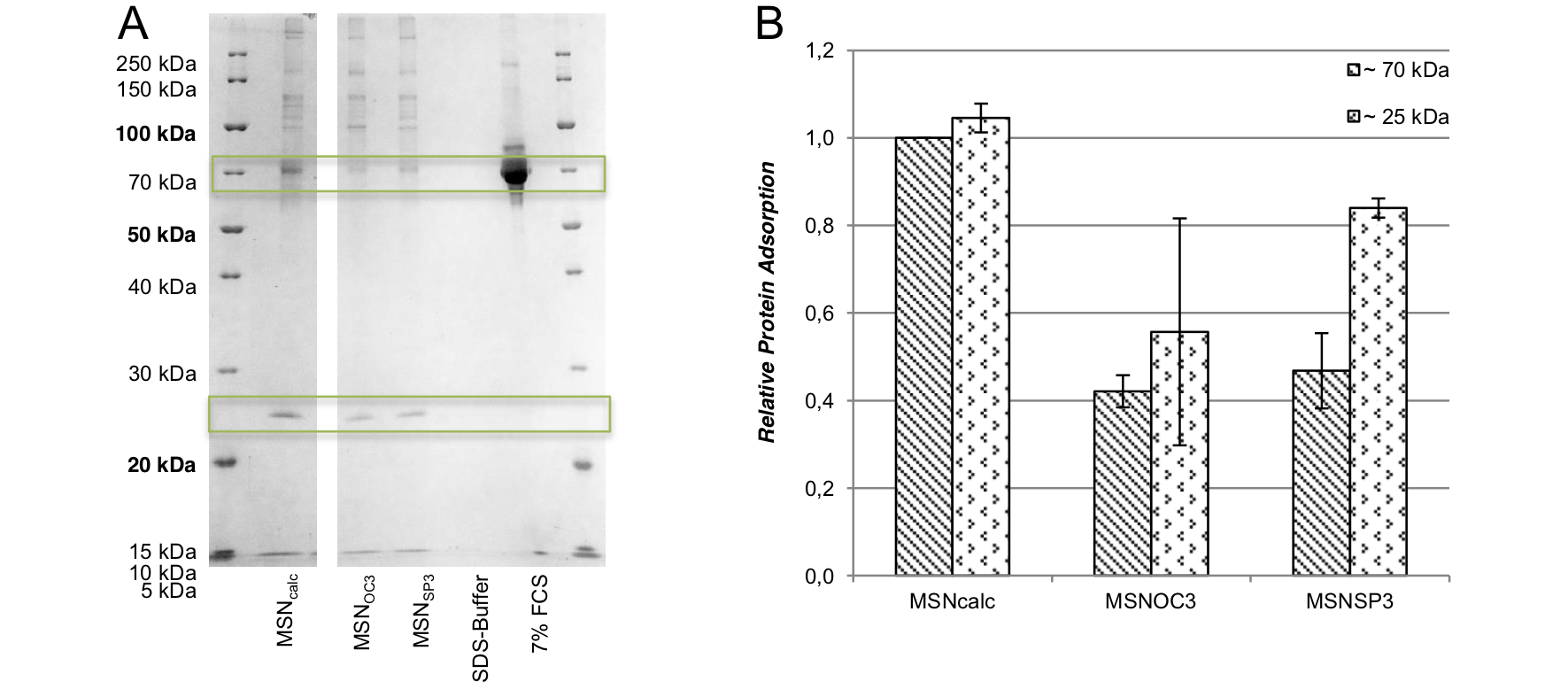


Figure S5. (A) Composition analysis of protein coronas of MSN, MSN_OC3_ and MSNSP_3_ after 60 min incubation at 37 °C in medium with 7.0% (w/v) of FCS. SDS-PAGE of corona preps with subsequent Coomassie staining. (B) Densitometric analysis of the two dominant bands of *ca* 70 and 25 kDa (boxed on panel A; ascribed to serum albumin and apolipoprotein A-I, respectively). Signals were normalized to 70 kDa band of MSNcalc (taken for 1.0); shown are average values +/- SEM from 2 experiments.

**
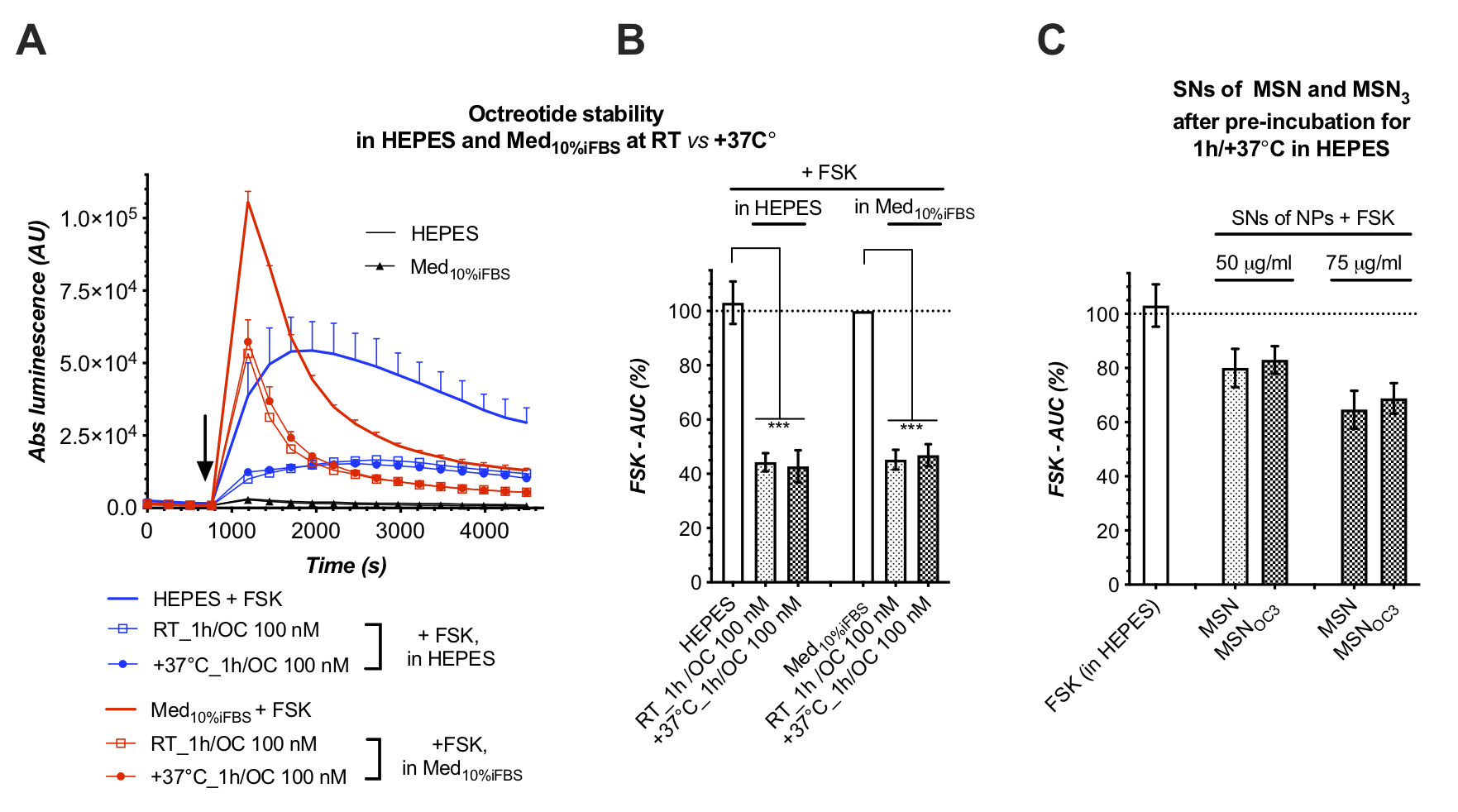
**

**Figure S6**. (**A/B**) OC maintains SSTR activating potency after 1h incubation at +37°C in both HEPES buffer (25 mM, pH 7.4) and DMEM/F12 medium with 10% iFBS, which indicates OC stability under these conditions. (**C**) Supernatants of non-capped MSNs and MSN_OC3_, harvested after 1h incubation at +37°C in HEPES (25 mM, pH 7.4), trigger the same cAMP response in the sensor cells, which excludes significant OC leakage from MSN_OC3_ under these conditions.

(**A**) Luminescence responses in the sensor cells in a single representative run in 3x technical replicates (error bars denote SD, with only SD`s upper half depicted; error bars for (RT_1h/OC 100 nM + FSK in Med_10%FBS_) are omitted for visual clarity); the moment of spiking is indicated with the black arrow. (**B/C**) Shown are average values (+/- SEM) of ≥3 independent experiment (each - in 3x technical replicates), integrated via AUC values and normalized to response to FSK, dissolved in Med_10%iFBS_ (taken for 100%).

Statistics - one-way repeated measures ANOVA with Tukey`s correction for multiple comparisons; all the comparisons with the significance level <0.05 are indicated with asterisks. All the assays were run with HEK-GS/SSTR2_HA cells at standard conditions, with IndMed_10%FBS_.

Note the clear difference in the dynamics of cAMP generation and decay between the sensor cells exposed to FSK, delivered in different media: once FSK is administered as solution in Med_10%FBS_ (panel A; red curves), intracellular cAMP rises faster, reaches higher levels and tends to decrease quicker afterwards as compared to cAMP response in cells receiving FSK in HEPES (panel A; blue curves), which is characterized by slower increment after stimulation, lower peak levels and less rapid decline. The molecular events behind these events remain poorly understood and could be speculatively attributed to transient effects of serum components on GPCR signaling upon spiking of FSK in Med_10%FBS_ to the pre-equilibrated medium, bathing the sensor cells.

Most importantly, the described effects are highly reproducible and have several practical implications. Firstly, the general pattern of cAMP response to MSNs in the sensor cells is «shaped» by the nature of medium MSNs and FSK are administered in. In line with this, MSNs with preformed corona, which are suspended in serum-enriched medium and mixed with FSK before spiking to the sensor cells, trigger «sharp» сAMP dynamics, i.e. quick and profound rise with subsequent steep luminescence decline that is characteristic of response to FSK after its pre-incubation in serum-containing medium. In contrast, HEPES-suspended MSNs normally exhibit «blunted» cAMP oscillations, similar to the ones described for FSK dissolved in HEPES (compare **Figures 6A** and **5A/C/E** for responses to MSNs with and w/o corona, respectively). Secondly, direct comparative AUC analysis of luminescent curves from samples prepared in different solvents could be misleading, for variation in the shapes of the curves under comparison is not fully accounted for. This could be exemplified with panels A and B of the present figure: luminescent curves of FSK in HEPES and medium are clearly distinct (A), yet the derived FSK-AUC% values fail to discriminate them as separate entities, as integral areas under the curves happen to be very close (B). With this, comparative AUC analysis of cAMP curves from samples in different media (cross-group) should be performed with caution and could only be justified once signals in the compared samples follow about the same pattern, which could be easily verified by visual inspection of luminescence plots. Intragroup comparisons of samples prepared in the same medium do not suffer from this limitation though (the majority of cases in the present work).
